# Efficacy of pH‐Responsive Surface Functionalized Titanium Screws in Treating Implant‐associated *S. aureus* Osteomyelitis with Biofilms Formation

**DOI:** 10.1002/adhm.202403261

**Published:** 2024-11-27

**Authors:** Hang Zhou, Youliang Ren, Kaixiong Zou, Ying Jin, Hang Liu, Haitao Jiang, Lei Shi, Xiaomin Sheng, Jason Weeks, Hannah Wang, Thomas Xue, Edward M. Schwarz, Chao Xie, Zhongliang Deng, Lin Wang, Lei Chu

**Affiliations:** ^1^ Department of Orthopaedics The Second Affiliated Hospital of Chongqing Medical University Chongqing 400010 P. R. China; ^2^ National Engineering Research Center for Tissue Restoration and Reconstruction South China University of Technology Guangzhou 510006 P. R. China; ^3^ Department of Orthopaedics New York Medical College New York NY 10595 USA; ^4^ Department of Orthopaedics, Center for Musculoskeletal Research University of Rochester Medical Center Rochester NY 14642 USA

**Keywords:** antimicrobial peptides, biofilm, implant‐associated *S. aureus* osteomyelitis, osteointegration, pH‐responsive

## Abstract

Implant‐associated *Staphylococcus aureus (S. aureus)* osteomyelitis (IASO) leads to high orthopedic implant failure rates due to the formation of *Staphylococcal* abscess community within the bone marrow and bacterial colonization in the osteocyte lacuno‐canalicular network (OLCN). To address this, antimicrobial peptides (HHC36)‐loaded titania nanotubes (NTs) are developed on titanium screws (Ti‐NTs‐P‐A), which integrate pH‐responsive polymethacrylic acid to control HHC36 release for eradicating bacteria in IASO. Colony‐forming unit assay confirmed that Ti‐NTs‐P‐A screws maintained sustainable antibacterial effectiveness, killing over 65% of *S. aureus* even after multiple bacterial solution replacements. Notably, Ti‐NTs‐P‐A screws exhibit significant pH‐responsive HHC36 release behavior and bactericidal activity, consistent with the phenotype of peptides‐killed bacteria from scanning electron microscopy. Transcriptome sequencing results reveal that Ti‐NTs‐P‐A screws interfered with ribosome formation and disrupted the arginine biosynthesis, which is crucial for bacterial survival in acidic environments. In the non‐infected implant model, the bone‐implant contact ratio of the Ti‐NTs‐P‐A screw is 2.3 times that of the clinically used titanium screw. In an IASO model, Ti‐NTs‐P‐A screws effectively eradicated bacteria within the OLCN, achieving an 80% infection control rate and desirable osteointegration. Collectively, Ti‐NTs‐P‐A screws with pH‐responsive antibacterial properties exhibit great potential for eradicating bacteria and achieving osseointegration in IASO.

## Graphical abstract

In the treatment of implant‐associated *S. aureus* osteomyelitis (IASO), antibiotics often fail to eradicate the bacteria in the osteocyte lacuno‐canalicular network, leading to a high implant failure rate. To overcome this, titanium screws are modified with antimicrobial peptides (HHC36)‐loaded titania nanotubes, which integrate pH‐responsive polymethacrylic acid (PMAA) to control HHC36 release for effective bacterial eradication and desirable osteointegration in IASO.

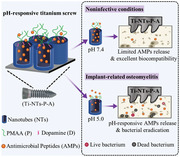

## 1 Introduction

Implant‐associated osteomyelitis (IAO) poses a severe challenge in orthopedic practice, often requiring multiple revisions and leading to poor functionality and outcomes.^[^ ^1^ ^]^ *Staphylococcus* aureus (*S. aureus*) is the main pathogen.^[^ ^2^ ^]^ Despite the widespread use of standard‐of‐care (SOC) treatments‐implant removal, surgical debridement, and systematic antibiotic outcomes for implant‐associated *S. aureus* osteomyelitis (IASO) have not significantly improved over the past decades.^[^ ^1^, ^3^ ^]^ Currently, residual bacteria within *Staphylococcus* abscess communities (SAC) and the osteocyte lacuno‐canalicular network (OLCN) are primarily responsible for post‐revision implant failures.^[^ ^4^ ^]^ These bacteria can survive from antibacterial therapy, causing catastrophic bone destruction and implant failure.^[^ ^5^ ^]^ The 2023 International Consensus Meeting on Musculoskeletal Infection emphasized that eradicating residual bacteria is crucial for the treatment of IAO.^[^ ^6^ ^]^

Systemic antibiotic therapy is often ineffective in eradicating the bacteria within OLCN due to the insufficient local concentration to reach the minimum inhibitory concentration (MIC) for bacteria.^[^ ^7^ ^]^ In contrast, local delivery of antibacterial agents from implants can achieve an adequate concentration of antibiotics at the target site, effectively combating bacteria while minimizing systemic toxicity in patients.^[^ ^8^ ^]^ This approach is crucial, as recent intravital microscopy studies in mice demonstrated that the race for the surface of the implant is completed by 3 h post‐operation.^[^ ^9^ ^]^ Consequently, various local antibacterial agent release systems have been developed for bone infection treatments, particularly in widely used orthopedic titanium‐based implants.^[^ ^10^ ^]^ Researchers have integrated metal elements, antibiotics, or antimicrobial peptides (AMPs) on the surface of titanium‐based materials to prevent biofilm formation and bacterial infection.^[^ ^10^, ^11^ ^]^ Notably, AMPs are proposed as one of the desired antibacterial agents due to their excellent broad‐spectrum bactericidal activity and low susceptibility to drug resistance, and they are able to combat bacteria in a “membrane disrupting” way.^[^ ^12^ ^]^ Additionally, titanium nanotubes (TiO_2_‐nanotubes, Ti‐NTs) have garnered attention for local AMPs release due to their unique topography and excellent loading capability, which could protect AMPs from enzyme‐mediated degradation during the initial stages of implantation.^[^ ^13^ ^]^

In order to prevent possible re‐infection from residual bacteria, on‐demand AMPs released from implants is valuable for IAO treatment. Namely, under noninfective conditions, AMPs release should be minimal to avoid potential cytotoxicity and prolong the antibacterial effectiveness of the implant. Conversely, in infective conditions, the implant should detect the infection and release sufficient AMPs to eradicate the bacteria. Our previous studies along with other reports have demonstrated that the acidic environment of bacterial infections can be employed to develop a pH‐responsive antibacterial surface on implants.^[^ ^10^, ^12^, ^14^ ^]^ Among these studies, the potential of pH‐responsive materials as the infection‐sensitive switch is systematically investigated.^[^ ^3^, ^10^, ^15^ ^]^ As reported, poly (methacrylic acid) (PMAA) displayed great advantages in the pH‐responsive release of antibacterial agents via switchable molecular conformational changes (swells in pH 7.4 and collapses in pH ≤ 6.0).^[^ ^12^, ^16^ ^]^

In our previous study, we successfully synthesized pH‐responsive PMAA‐gated TiO_2_ nanotubes on titanium (Ti) plates, achieving on‐demand AMPs release through the “swelling‐collapsing” transformation of PMAA molecules. After loading with the antimicrobial peptide HHC36, these PMAA‐gated nanotubes could release HHC36 at a slow rate to prevent latent bacterial infection and maintain biocompatibility under physiological conditions, with a burst release occurring once bacterial infection occurs.^[^ ^12c^ ^]^ While this technology effectively controls AMPs release and provides long‐lasting antimicrobial effects, there remained a potential risk of nanotube detachment when applied in real scenarios, such as on bone screws that require high torque. This risk significantly limited the clinical application of this technology.

**Scheme 1:**
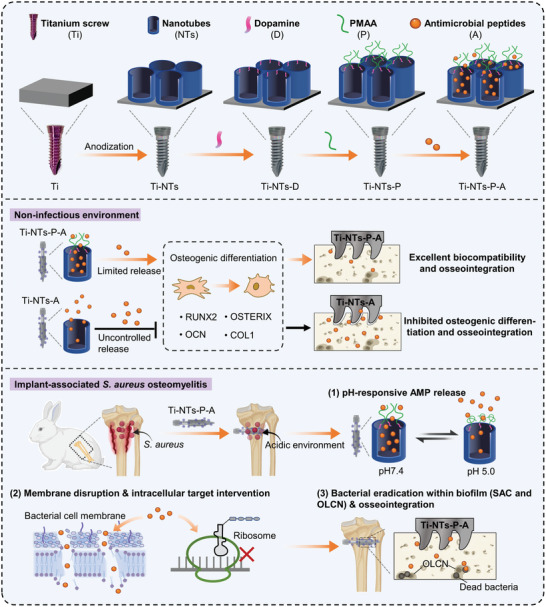
Design of the titanium screws modified with pH‐responsive TiO_2_ nanotubes for on‐demand release of antimicrobial peptides and enhanced implant osseointegration in implant‐associated *S. aureus* osteomyelitis.

In this study, we aimed to enhance the stability of the nanotubes and validate the application of pH‐responsive nanotubes on Ti screws for on‐demand AMPs release and osseous integration in IASO (**Scheme** **1**). We chose commercial titanium screws and prepared enhanced TiO_2_ nanotubes on them, along with immobilizing PMAA and loading HHC36 (named Ti‐NTs‐P‐A screws). In the acidic infection environment, this Ti‐NTs‐P‐A screw exhibited significant pH responsiveness and enhanced antibacterial ability through on‐demand release of HHC36. Simulating the clinical implantation process, we demonstrated that Ti‐NTs‐P‐A screws maintained their pH‐responsive antibacterial properties despite mechanical stress. To investigate the bactericidal activity of these screws and possible potential mechanisms, we performed colony forming units (CFU) assays, scanning electron microscope (SEM), and transcriptome‐sequencing analysis. Importantly, the bacterial eradication and osteointegration of Ti‐NTs‐P‐A screws were investigated in a well‐established one‐stage revision implant‐associated *S. aureus* osteomyelitis (IASO) model. Additionally, under noninfective conditions, both in vitro and in vivo experiments were investigated to analyze the biocompatibility and osteointegration of these screws.

## 2 Results

### 2.1 The Preparation and pH‐Activated HHC36 Release of the Functionalized Screws

**Figure 1:**
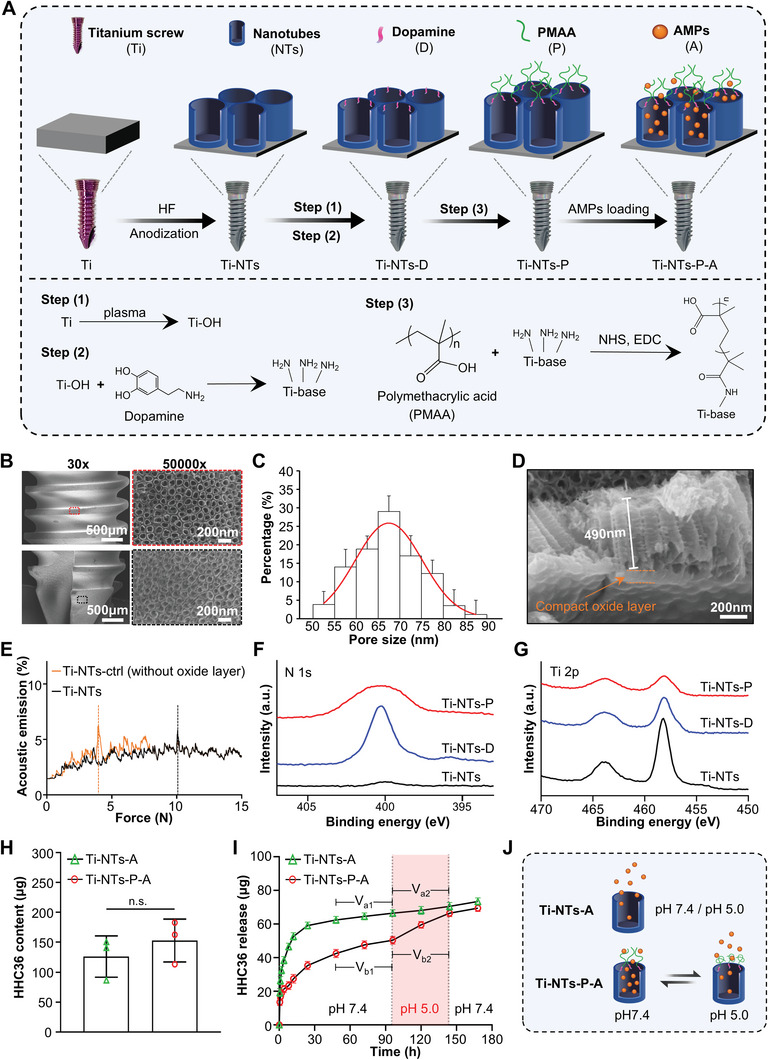
The preparation and pH‐activated AMPs (HHC36) release of the functionalized titanium screws. A) Schematic illustrating the preparation process of functionalized titanium screws, which were modified with PMAA‐gated and AMPs‐loading TiO_2_ nanotubes. B) SEM images and C) pore size distribution of the TiO_2_ nanotubes on the Ti‐NTs screws. D) The SEM image of a cross‐sectional view of TiO_2_ nanotubes with a compact oxide layer. E) Curves of acoustic signal and frictional force during the scratch test. F) High‐resolution XPS N 1s and G) Ti 2p spectra of the indicated screws exhibited the immobilization of dopamine and PMAA molecules. H) AMPs loading content of the Ti‐NTs‐A and Ti‐NTs‐P‐A screws (*n* = 3). I) HHC36 released from the Ti‐NTs‐A and Ti‐NTs‐P‐A screws (*n* = 3), where the pH of the PBS solution was adjusted from 7.4 to 5.0 for the 96–144 h period. Specifically, the average HHC36 release rates 48 h before and after pH change (from 7.4 to 5.0) were denoted as V_a1_ and V_a2_ in the Ti‐NTs‐A group, and as V_b1_ and V_b2_ in the Ti‐NTs‐P‐A group, respectively. J) Illustration of the mechanism of pH‐activated AMPs release of the Ti‐NTs‐P‐A screw. At pH 7.4, PMAA molecules swell, limiting AMPs release by obstructing the nanotubes. When the pH value drops to 5.0, PMAA molecules collapse to open the nanotubes, rapidly releasing AMPs. Data are shown as the mean ± SD, n.s. represents no significance. AMPs, antimicrobial peptides; PMAA, polymethacrylic acid; NHS, N‐hydroxysuccinimide; EDC, 1‐ethyl‐3‐(3‐dimethylaminopropyl)‐carbodiimide hydrochloride.

A schematic illustration of the preparation process of functionalized titanium screws (Ti‐NTs‐P‐A) is shown in **Figure** **1**A. In brief, the enhanced TiO_2_ nanotubes on titanium screws were prepared via double anodization and gated with pH‐responsive PMAA molecules for the on‐demand release of AMPs as previously reported.^[^ ^12^, ^17^ ^]^ As shown in Figure 1B–D, uniform TiO_2_ nanotubes formed on the prepared Ti‐NTs screws after anodization, and the average pore size and length of TiO_2_ nanotubes was approximately 67 and 490 nm, respectively. These TiO_2_ nanotube arrays were considered to have great potential for the surface functionalization and drug loading of Ti‐based implants, while the weak adhesion of TiO_2_ nanotubes to the underlying Ti substrate compromises many promising applications.^[^ ^18^ ^]^ To address this problem, we conducted additional anodization and synthesized a ≈100 nm thick compact oxide layer between the TiO_2_ nanotubes and Ti substrate (Figure 1D).^[^ ^17^ ^]^ The scratch tests (Figure 1E) showed that the prepared Ti‐NTs have a higher L_c_ value of ≈10 N compared with that of Ti‐NTs‐ctrl (without compact oxide layer, ≈4 N), indicating the creased adhesion of the TiO_2_ nanotubes coating after the double anodization. X‐ray photoelectron spectroscopy (XPS) was employed to exhibit the immobilization process of dopamine and PMAA molecules on the screws (Figure 1F,G). Compared to Ti‐TNs, the specific N 1s peak appeared and the Ti 2p signal was reduced on Ti‐NTs‐D after dopamine grafting, indicating the successful immobilization of dopamine molecules. After the PMAA grafting process, a decreased N 1s peak was observed on Ti‐NTs‐P compared with that of Ti‐NTs‐D, suggesting the immobilization of PMAA molecules. Moreover, the conjugation of PMAA with dopamine was further demonstrated by the changes in the atom ratios between elements (Figure S1, Supporting Information). For AMPs loading, the pH of the HHC36 solution was adjusted to 5.0 to “open” the PMAA gates. As Figure 1H shows, no significant difference in AMPs loading content was observed between Ti‐NTs‐A (127.0 ± 32.8 µg) and Ti‐NTs‐P‐A (153.3 ± 26.5 µg) (*p* > 0.05) groups, indicating the dopamine and PMAA immobilization process did not impact the loading capability of TiO_2_ nanotubes.

Next, we measured the rate of HHC36 release from Ti‐NTs‐A and Ti‐NTs‐P‐A screws in PBS solution, and during the release process, the pH value of the solution was adjusted from 7.4 to 5.0 to simulate an acidic infectious environment. As Figure 1I shows, an initial burst release was observed both in the Ti‐NTs‐A and Ti‐NTs‐P‐A groups (0–8 h) in PBS with a pH of 7.4. Notably, this burst release is highly necessary in orthopedic clinical settings to eliminate various bacteria introduced during surgical procedures as well as endogenous bacteria present in the bone marrow cavity.^[^ ^19^ ^]^ Once the initial release stabilized, decreased HHC36 release was observed in the Ti‐NTs‐P‐A group (50.43 ± 3.51 µg) compared with the Ti‐NTs‐A group (66.49 ± 3.72 µg) (*p* < 0.01). Critically, a significantly increased HHC36 release rate was observed in the Ti‐NTs‐P‐A group when pH decreased (V_b1_, 0.17 ± 0.00 µg h^−1^; V_b2_, 0.34 ± 0.00 µg h^−1^) (*p* < 0.001), while no obvious change of the HHC 36 release rate was noted in Ti‐NTs‐A group (V_a1_ = V_a2_, 0.08 ± 0.01 µg h^−1^) (*p* > 0.05). Specifically, within the low pH‐triggered 48 h release period, the AMPs release from the Ti‐NTs‐P‐A group (21.62 ± 0.21 µg) reached its MIC value (18.5 ug mL^−1^). In sum, we observe that after the initial release stabilizes, a pH change can further trigger sufficient AMPs release from the Ti‐NTs‐P‐A screw to inhibit bacteria effectively. Conversely, due to excessive initial burst release, the AMPs release from Ti‐NTs‐A (5.48 ± 0.46 µg) failed to reach the MIC value within the low pH‐triggered 48 h release period, thus unable to effectively suppress infection in the long term (Figure 1J).

### 2.2 In Vitro and Ex Vivo Evidence of pH‐Activated Antibacterial Properties of the Functionalized Screws

**Figure 2:**
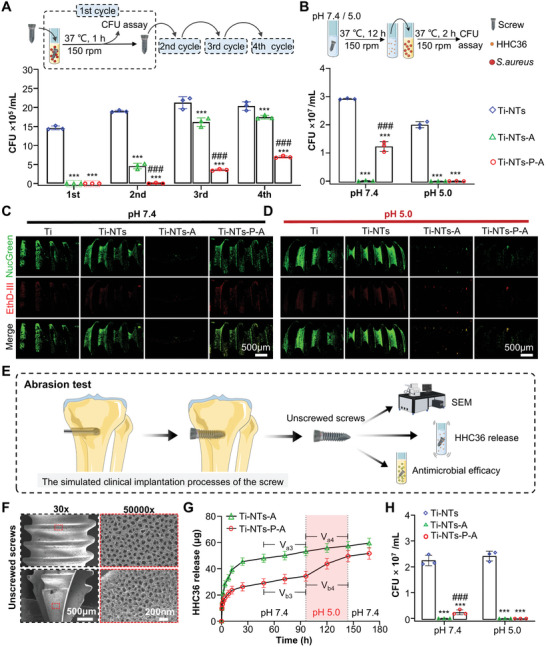
In vitro and ex vivo evidence of pH‐activated antibacterial properties of the functionalized titanium screws. A) The antibacterial activity of the indicated screws against *S. aureus* cultures for 1h, and for four cycles (*n* = 3). B) The antibacterial activity of the soaking solution of the indicated screws at pH 5.0 and pH 7.4 against *S. aureus*, was assessed by CFU (*n* = 3). C,D) Representative 3D reconstructed fluorescence images of live/dead stained *S. aureus* after incubation with the indicated screws for 4h (Green, live, and dead bacteria; Red, dead bacteria). E) The friction stabilities of functionalized screws were tested by screwing them into the bone, unscrewing them, and then examining their morphology and function. The friction due to implantation did not affect the physical properties of Ti‐NTs‐P‐A, as shown by representative SEM images in F) (top: screw body; bottom: screw tip). G) The HHC36 release from the unscrewed Ti‐NTs‐A and Ti‐NTs‐P‐A screws (*n* = 3), where the pH of the PBS solution was adjusted from 7.4 to 5.0 for the 96–144 h period. Specifically, the average HHC36 release rates 48 h before and after the pH change (from 7.4 to 5.0) were denoted as V_a3_ and V_a4_ in the Ti‐NTs‐A group and denoted as V_b3_ and V_b4_ in the Ti‐NTs‐P‐A group, respectively. H) The antibacterial activity of the soaking solution of the unscrewed screws at pH 5.0 and pH 7.4 against *S. aureus* (*n* = 3). The data are shown as the means ± SDs, ^***^ *p* < 0.001, in comparison with the Ti‐NTs group, ^###^ *p* < 0.001 in comparison with the Ti‐NTs‐A group.

The Ti‐NTs‐P‐A screws showed a more stable antibacterial activity than Ti‐NTs‐A screws in the multi‐cycle test (**Figure** **2**A). Ti‐NTs‐P‐A killed 83% and 65% of bacteria in the third and fourth cycles, respectively, compared to Ti‐NTs‐A which killed only 24% and 14%. As Figure 2B shows, the antibacterial properties of the prepared screws against *S. aureus* at different pH were characterized, and Ti‐NTs without antibacterial activity were used as control. Ti‐NTs‐A exhibited equally outstanding antibacterial ability both at pH 7.4 and 5.0, while Ti‐NTs‐P‐A displayed pH‐responsive bactericidal activity. At pH 7.4, the bactericidal activity of Ti‐NTs‐P‐A was relatively weak, inhibiting 57.8% of bacterial growth. Notably, this limited antibacterial activity of Ti‐NTs‐P‐A is expected to prevent biofilm formation in a physiological environment (pH 7.4) with negligible cytotoxicity.^[^ ^15^, ^20^ ^]^ At pH 5.0, Ti‐NTs‐P‐A presented excellent antibacterial activity comparable to Ti‐NTs‐A, which could be attributed to the increased HHC36 release from opened nanotubes as mentioned above. As the 3D reconstructed fluorescence images of live/dead stained *S. aureus* (Figure 2C,D) show, live *S. aureus* adhered abundantly to the surfaces of Ti and Ti‐NTs screws, indicating that the Ti and Ti‐NTs lacked antibacterial ability. The pH‐activated antibacterial ability of Ti‐NTs‐P‐A was further demonstrated with significantly decreased live bacteria adhesion at pH 5.0 compared with pH 7.4.

In consideration of the clinical application of orthopedic screws, the friction stability of functionalized screws was characterized following the schematics in Figure 2E. Representative SEM images (Figure 2F) and corresponding quantitative analysis (Figure S2, Supporting Information) showed negligible change in the morphology of the TiO_2_ nanotubes after implantation. In addition, the pH‐activated HHC36 release (Figure 2G) of post‐implanted Ti‐NTs‐P‐A was retained, suggesting that the functionalized surface was resistant to the implantation process. The Ti‐NTs‐P‐A screw could still kill 99% of bacteria (Figure 2H; Figure S3, Supporting Information), demonstrating that it is a reasonable candidate for in vivo application.

### 2.3 Evaluation of the Antibacterial Properties of the Functionalized Screws by SEM

**Figure 3:**
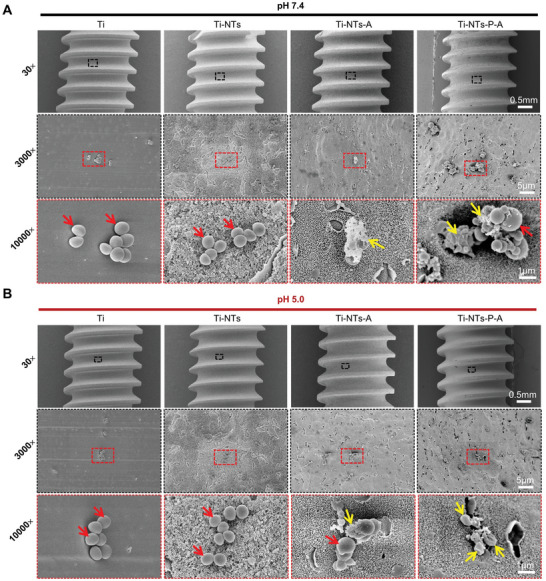
Bacterial membrane disruption caused by the functionalized screws. SEM images of *S. aureus* after cultured with the functionalized screws at A) pH 7.4 or B) pH 5.0 for 4 h. Red arrows indicate normal‐growing *S. aureus* with round and smooth appearance. Yellow arrows indicate the dead bacteria with deformed or ruptured membranes.

To better understand the bactericidal activity of Ti‐NTs‐P‐A screws, the morphologic change of *S. aureus* cultured with different screws was evaluated via SEM. Representative SEM images demonstrated that at pH 7.4 and pH 5.0, uniformly‐sized *S. aureus* (red arrow) with spherical and smooth membranes were present on the surface of Ti and Ti‐NTs screws (**Figure** **3**), indicating that these screws lack antibacterial activity. In contrast, at pH 7.4, obvious bacterial membrane fragmentation, rupture, and cell lysis were observed in the Ti‐NTs‐A and Ti‐NTs‐P‐A groups (yellow arrow) (Figure 3A). Specifically, in the Ti‐NTs‐P‐A treated group, many shrunken bacteria (yellow arrows) and irregular particles of different sizes adjected to the bacterial colonies (red arrow) were observed. These substances were considered to be metabolites released from the HHC36‐killed bacteria.^[^ ^21^ ^]^ Furthermore, in the pH 5.0 environment, more pronounced bacterial membrane deformation and rupture, as well as significantly reduced bacterial adhesion, were observed in the Ti‐NTs‐P‐A group compared to pH 7.4 (Figure 3B; Figure S4, Supporting Information). These findings confirm that Ti‐NTs‐P‐A screws exhibit remarkable pH‐responsive antibacterial properties and can kill *S. aureus* by inducing the destruction of the bacterial cell membrane.

### 2.4 Transcript Profile Analysis of the *S. aureus* Treated with the Functionalized Screws

**Figure 4:**
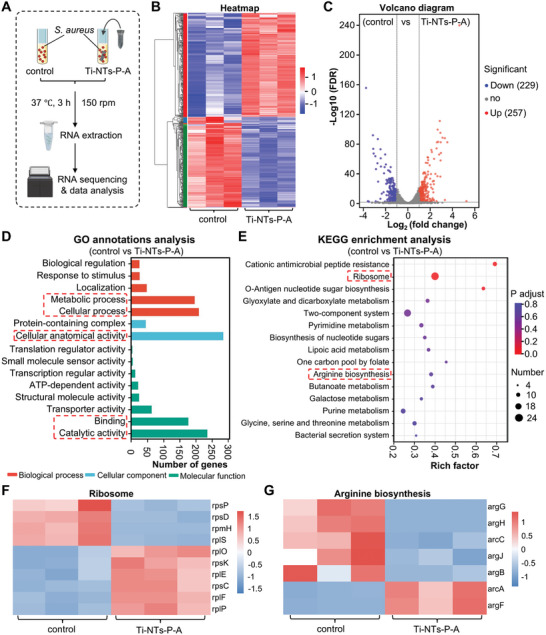
Transcriptome analysis of the *S. aureus* after cultured with or without the Ti‐NTs‐P‐A screw for 3 h. A) Schematic diagram of the RNA sequence analysis of the Ti‐NT‐P‐A and control groups. B) Heatmap representing the top 100 genes significantly differentially expressed between the Ti‐NT‐P‐A group and the control group. C) Volcano plot of differentially expressed genes. The green dots and red dots indicate upregulated and downregulated differentially expressed genes, respectively. The grey dots represent nonsignificantly expressed genes. D) GO annotations and E) KEGG enrichment analyses of differentially expressed genes (*p* value < 0.05). Heatmap of the differentially expressed genes related to F) ribosome (top 10 DEGs) and G) arginine biosynthesis.

In addition to membrane‐targeting bactericidal activity, AMPs have been reported to act on other intracellular targets.^[^ ^12^, ^22^ ^]^ In order to reveal the potential antibacterial mechanisms of the Ti‐NTs‐P‐A screws, transcriptome sequencing techniques were employed to detect the transcript profile changes in *S. aureus* induced by the HHC36‐loading Ti‐NTs‐P‐A screws (**Figure** **4**A). The heatmap and volcano diagram showed that *S. aureus* exhibited 486 significantly differentially expressed genes (DEGs) following treatment with the Ti‐NTs‐P‐A screw for 3 h (Figure 4 B,C). Among these, 229 genes were downregulated, and 257 genes were upregulated. Gene Ontology (GO) analysis indicated that these DEGs were mostly associated with metabolic process, cellular process, cellular anatomical activity, binding, and catalytic activity (Figure 4D). Additionally, the Kyoto Encyclopedia of Genes and Genomes (KEGG) annotation analysis showed that these DEGs were involved in the translation, membrane transport, signal transduction, and the metabolism of amino acids and carbohydrates (Figure S5, Supporting Information). KEGG enrichment analysis (Figure 4E) revealed that pathways associated with ribosome, pyrimidine metabolism, and arginine biosynthesis were significantly impacted. The heatmap of DEGs involved in the ribosome showed that the genes associated with the synthesis of ribosomal subunits (such as rpsP and rpmH) were markedly downregulated, indicating Ti‐NTs‐P‐A could affect the ribosome assembly and cellular protein biosynthesis.^[^ ^23^ ^]^ (Figure 4F). It is well‐known that the arginine system is required for bacterial growth in an acidic environment, whereas the absence of arginine can lead to citrate cycle disturbance, accumulation of ROS, and DNA damage.^[^ ^24^ ^]^ In our study, the expression of multiple genes associated with arginine biosynthesis, including argG, argH, arcC, argJ, and argB, was significantly downregulated (Figure 4G), suggesting that the Ti‐NTs‐P‐A treatment could interfere with arginine biosynthesis. Notably, arcC is an essential gene for the arginine‐deiminase system (Arc), which mediates the tolerance of *S. aureus* to the acidic environment, the downregulation of arcC could affect bacterial survival in an acidic environment and within the host.^[^ ^25^ ^]^ These results suggest that HHC36 release from Ti‐NTs‐P‐A screws displays a multifaceted antibacterial mechanism against *S. aureus*. Collectively, HHC36 may not only destroy the integrity of the bacterial membrane but also cause comprehensive changes to translation, amino acid metabolism, and other functions.

### 2.5 In Vitro Cytocompatibility and Osteoinductive Capacity of the Functionalized Screws

**Figure 5:**
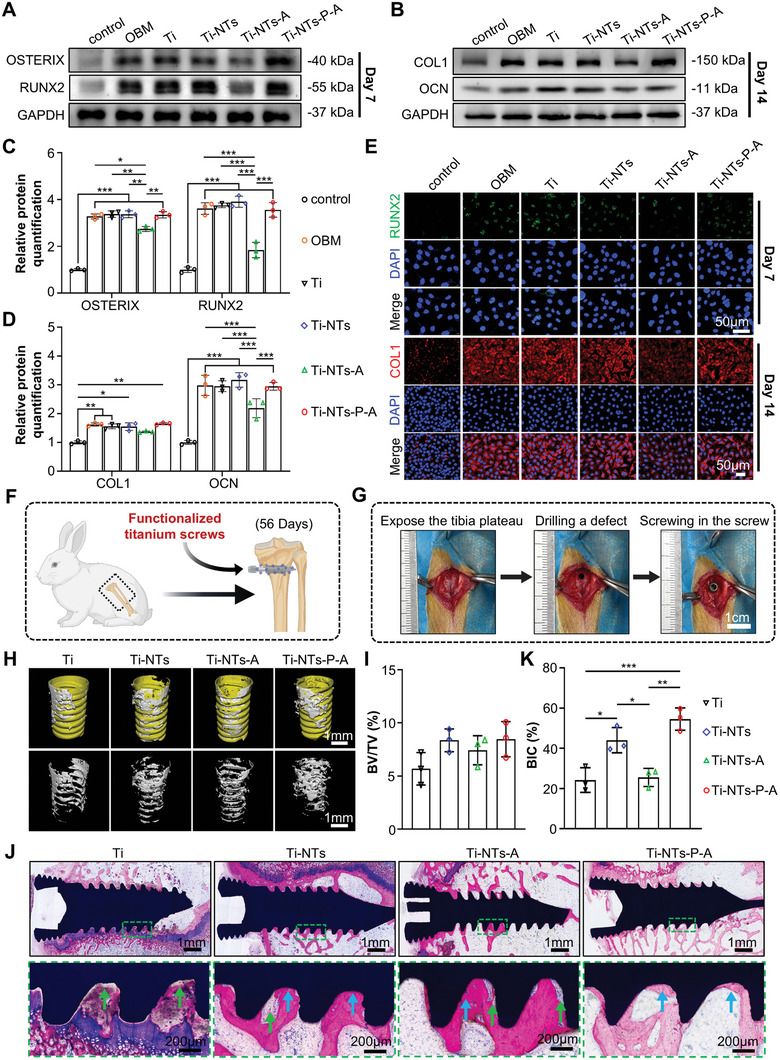
In vitro and in vivo biocompatibility and osseointegration evaluation of the functionalized titanium screws. A,B) Representative western blot images of the expression of osteogenesis‐related proteins and C,D) corresponding quantitative analyses (*n* = 3). E) Immunofluorescence images of RUNX2 and COL1 staining of MEFs in different groups. F) Schematic diagram of the osseointegration and biocompatibility test of functionalized screws in an uninfected bone defect model. G) The implantation process of functionalized screws in rabbit tibial plateau. H) The micro‐CT 3D reconstructed images and I) bone tissue volume/total tissue volume (BV/TV) analysis from micro‐CT results of the newly formed bone surrounding the screws after 56 days of implantation (*n* = 3). J) The methylene blue‐basic magenta staining and corresponding K) bone‐implant contact (BIC) ratio analysis of the non‐decalcified bone sections for osseointegration assay (*n* = 3). Green arrows indicate gaps between the screw and bone tissue. Blue arrows indicate bone‐implant contact areas. In I,J), The data are shown as the means ± SDs, ^*^ *p* < 0.05, ^**^ *p* < 0.01, ^***^ *p* < 0.001.

Multipotent mouse embryonic fibroblasts (MEFs) were used to evaluate the cytotoxicity and osteogenic properties of the functionalized screws.^[^ ^26^ ^]^ The Cell Counting Kit‐8 (CCK‐8) assay reveals that the incubation medium from the Ti‐NTs‐A and Ti‐NTs‐P‐A screws display no discernible effects on MEFs proliferation and both groups show similar cell viability to control groups, suggesting these HHC36‐containing medium exhibited good cytocompatibility (Figure S6, Supporting Information). The alkaline phosphatase (ALP) activity is an essential indicator of early osteogenesis.^[^ ^27^ ^]^ As shown in Figure S7 (Supporting Information), the ALP activity of MEFs was significantly increased in the OBM group in comparison with the control group, indicating a successful induction of osteogenic differentiation. Compared to the OBM group, the ALP activity of MEFs presented no statistically significant changes in the Ti, Ti‐NTs, and Ti‐NTs‐P‐A groups, while exhibiting a stark decrease in the Ti‐NTs‐A group, suggesting uncontrolled HHC36 release from Ti‐NTs‐A screws may lead to inhibited osteogenic differentiation of MEFs. We further detected the osteogenic differentiation‐related protein expression of MEFs, including early markers (RUNX2 and OSTERIX) and late markers (COL1 and OCN) of osteogenesis (**Figure** **5**A–D). The expressions of RUNX2, OSTERIX, and OCN were significantly suppressed in the Ti‐NTs‐A group compared with the OBM group, indicating the uncontrolled AMP lease from Ti‐NTs‐A screws would impact the osteogenic differentiation of MEFs. Interestingly, no significant change in the expression of the markers of osteogenesis was found in the Ti‐NTs‐P‐A group in comparison with the OBM group. These findings were further demonstrated by the immunofluorescence staining of MEFs (RUNX2 and COL1) (Figure 5E; Figure S8, Supporting Information). The above results reveal that grafting TiO_2_ nanotubes with PMAA molecules can effectively avoid the inhibition of osteogenic differentiation caused by the uncontrolled release of HHC36, significantly improving the in vitro biocompatibility of the Ti‐NTs‐P‐A screws.

### 2.6 In Vivo Osseointegration of the Functionalized Screws in Non‐Infected Model

In this study, a sterilized implant rabbit model was applied to assess the osseointegration and biocompatibility of the prepared screws (Figure 5F,G). As shown in the micro‐CT results (Figure 5H,I; Figure S9, Supporting Information), after 8 weeks of implantation, newly formed bone surrounding screws was observed in all four groups, indicating good biocompatibility. Quantitative results indicated that, compared to pristine Ti, Ti‐NTs exhibited an increase in bone formation, suggesting that the nanotubes themselves had a bone‐promoting effect.^[^ ^28^ ^]^ After loading with HHC36, the bone formation in the Ti‐NTs‐A group decreased, which may be attributed to the toxicity caused by the burst release of HHC36.^[^ ^29^ ^]^ However, in the Ti‐NTs‐P‐A group, where the release of HHC36 was effectively controlled, the bone formation was increased compared to Ti‐NTs‐A and was similar to that of Ti‐NTs. The methylene blue‐basic magenta staining (Figure 5J) and bone‐implant contact (BIC) results (Figure 5K) suggested significantly increased osseointegration in the Ti‐NTs‐P‐A group compared to the Ti‐NTs‐A group, with less fibrous tissue gap between screw and bone tissue. These findings also demonstrate the importance of controlled release of AMPs in maintaining the biocompatibility of implants. Moreover, the serological analysis (Table S1, Supporting Information) and H&E staining of major organs (Figure S10, Supporting Information) of experiment rabbits suggested Ti‐NTs‐P‐A screw was biocompatible for in vivo application. In conclusion, the Ti‐NTs‐P‐A screw exhibits excellent osseointegration and biocompatibility in vivo, giving it great potential for clinical application.

### 2.7 In Vivo Antibacterial Ability and Osseointegration of the Functionalized Screws in Implant‐Associated *S. aureus* Osteomyelitis

**Figure 6:**
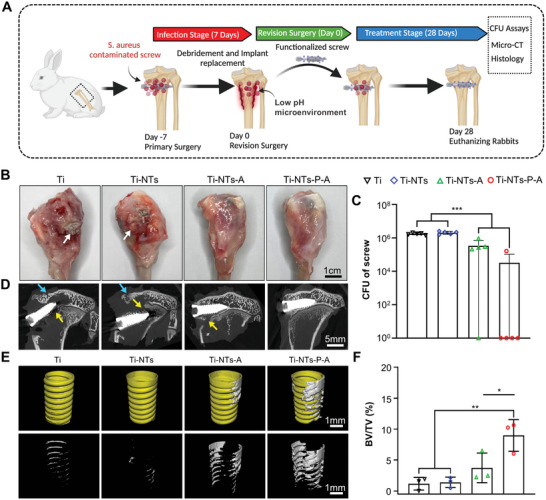
Radiographic and bacteriological assay of the therapeutic effect of the functionalized titanium screws in implant‐associated osteomyelitis. A) Schematic illustration of a rabbit 1‐stage revision model for implant‐associated *S. aureus* osteomyelitis. A Ti screw was contaminated in *S. aureus* suspension overnight, air‐dried for 30 min, and implanted in a rabbit's left tibia. 7 days after the implantation, the screw was removed followed by debridement and irrigation, and a sterile screw with or without functionalized surface was implanted for another 28 days. B) Representative images of harvested rabbit tibia 28 days post‐revision. White arrows indicated the purulent mass. C) The in vivo antibacterial activity of the indicated screws via CFU assay (*n* = 5). D) The reconstructed micro‐CT images of rabbit tibia 28 days post‐revision. Blue arrows indicate the infectious bone destruction of subchondral bone. Yellow arrows indicated the infectious bone destruction and collapse of the growth plate of the tibia. E) The micro‐CT 3D reconstructed images and corresponding F) BV/TV analysis of the newly formed bone surrounding the screws (*n* = 3). The data are shown as the means ± SDs, ^*^ *p* < 0.05, ^**^ *p* < 0.01, ^***^ *p* < 0.001.

Herein, we applied an established rabbit tibia 1‐stage revision model to assess the osseointegration of functionalized titanium screw in infectious conditions (**Figure** **6**A; Figure S11, Supporting Information).^[^ ^5^, ^30^ ^]^ After a 7‐day infection phase, the CFU assay (Figure S12, Supporting Information) of the unscrewed screws suggested abundant bacteria (1.7 × 10^6^ ± 4.0 × 10^5^ CFU) adhered on the implant. Moreover, similar biofilm bacteria were also observed among the sequestrum and trabecular space after 7 days of implantation (Figure S13, Supporting Information), demonstrating the successful establishment of an implant‐associated osteomyelitis model.^[^ ^4^, ^31^ ^]^

As shown in Figure 6B, obvious signs of inflammation and infection (purulent mass formation) were observed around the implant site in Ti and Ti‐NTs groups after 28 days post‐revision, while the Ti‐N‐A and Ti‐N‐P‐A group exhibited smooth tissue healing surrounding the implant site. The CFU assay (Figure 6C) of the unscrewed screws indicated significantly decreased CFUs in Ti‐N‐A and Ti‐NTs‐P‐A groups compared with that of Ti and Ti‐NTs groups, suggesting reduced bacteria adjacent to the implants. Interestingly, in the Ti‐NTs‐P‐A group, there was a 98% reduction in bacteria compared with that of the Ti group, which was even higher than the 81% reduction observed in the Ti‐NTs‐A group. We attribute this to the longer‐lasting controlled release of HHC36 from the Ti‐NTs‐P‐A, which allowed for a sustained concentration of HHC36 above the MIC, effectively suppressing bacterial growth.^[^ ^20^, ^32^ ^]^

The micro‐CT images (Figure 6D) show extensive infectious bone destruction and collapse of the growth plate of tibiae in Ti and Ti‐NTs groups. In addition, dislocation of screws was observed in both groups, indicating implant failure post‐revision. Although implant loosening was not observed in the Ti‐NTs‐A group, obvious destruction of cortical bone and growth plate was observed. This could be because the limited antibacterial property of Ti‐NTs‐A in the later stage of implantation could not inhibit the recurrence of infection. Notably, the Ti‐NTs‐P‐A screw was well‐fixed in the tibial plateau via osteointegration, showing outstanding antibacterial ability in infectious conditions. Furthermore, the quantitative analyses of the newly formed bone surrounding the screws (Figure 6E,F) indicated that Ti‐NTs‐P‐A had the highest bone volume compared to other groups, which was consistent with the radiographic results.

**Figure 7:**
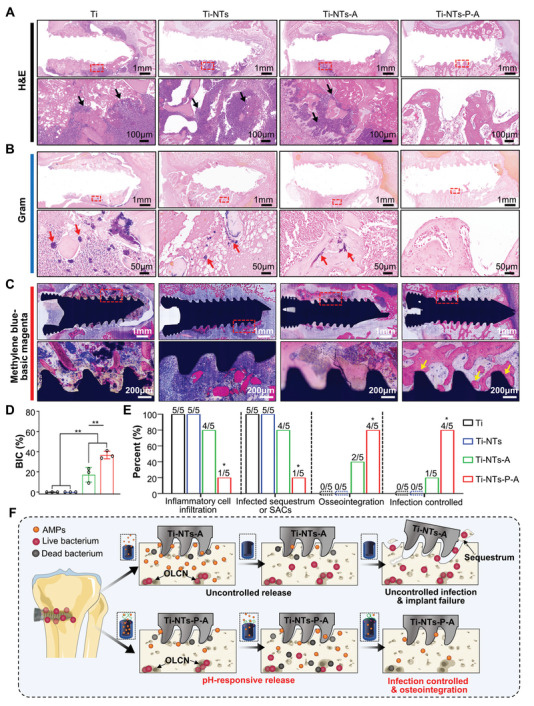
Histology illustrates the therapeutic effect of the functionalized titanium screws in implant‐associated osteomyelitis. A) Representative images of H&E staining of decalcified rabbit tibiae. Black arrows indicate inflammatory cells. B) Representative images of Gram staining of decalcified rabbit tibiae. Red arrows indicated Gram‐positive bacteria. C) Representative images of methylene blue‐basic magenta staining of undecalcified bone sections. Yellow arrows indicate the bone‐implant contact area. D) The BIC ratios of indicated screws after 28 days of implantation (*n* = 3). E) Histologic outcomes of indicated screws after 28 days of implantation. F) Diagram of the proposed mechanism by which functionalized titanium screws (Ti‐NTs‐P‐A) exhibit pH‐responsive antibacterial properties for bacterial eradication and osteointegration in implant‐associated osteomyelitis. The data are shown as the means ± SDs, ^*^ *p* < 0.05, ^**^ *p* < 0.01.

The antibacterial abilities and osseointegration of Ti‐N‐A and Ti‐NTs‐P‐A screws were further investigated through histologic staining and quantitative analysis (**Figure** **7**). H&E staining (Figure 7A) revealed extensive bone destruction and abundant inflammatory cell infiltration in the Ti and Ti‐NTs groups (black arrows), indicating that the wound debridement and irrigation could not eradicate the bacteria from the infected bone marrow completely. Similar histological characteristics could be observed in the Ti‐NTs‐A group (4 out of 5 samples), while only one case in the Ti‐NTs‐P‐A group (1 out of 5 samples) showed inflammatory cell and fibrous hyperplasia around screws. Gram staining (Figure 7B) revealed abundant Gram‐positive *S. aureus* within SAC and OLCN (red arrows) colonizing within the bone marrow cavity in the Ti and Ti‐NTs groups. In contrast, relatively few bacteria were observed within the necrotic bone fragments in the Ti‐NTs‐A group, suggesting that some bacteria could survive the initial HHC36 release and pose a high risk of bone infection recurrence.^[^ ^1^, ^33^ ^]^ Crucially, no Gram‐stained positive bacteria were observed in most of Ti‐NTs‐P‐A screws treated tibiae (4 out of 5 samples), indicating that the released HHC36 completely eradicated residual bacteria within SAC and OLCN. Interestingly, methylene blue‐basic magenta staining of the hard tissue sections demonstrated excellent osseointegration surrounding the functionalized screws in the Ti‐NTs‐P‐A treated *S. aureus*‐infected tibiae (Figure 7C) (yellow arrows). In the Ti and Ti‐NTs groups, large sequestrate surrounded these screws, with no osteointegration. Although some peri‐implant bone tissue retained normal structure in a few cases, extensive bone destruction surrounding the screw was observed in the Ti‐NTs‐A group, suggesting that *S. aureus* bone infection was not controlled well. Notably, in the Ti‐NTs‐P‐A treated tibiae, even though small areas of suspected bone destruction existed surrounding the screw, most of the bone surrounding the screw remained with normal structure. More importantly, quantitative analysis showed that the best osseointegration was achieved in the Ti‐NTs‐P‐A group compared to the other three groups according to the BIC results (Figure 7D). Based on the histologic evidence after 28 days of treatment with different screws (Figure 7E), it is clear that the Ti‐NTs‐P‐A screws exhibited excellent osseointegration and the highest rate of infection control.

The above results indicate that the Ti‐NTs‐P‐A screw, with its pH‐responsive AMPs release, demonstrates superior antibacterial ability in this well‐established implant‐associated *S. aureus* osteomyelitis animal model, while also achieving desired osteointegration (Figure 7F).

## 3 Discussion

Despite significant strides in antimicrobial therapies and surgical techniques, IAO remains a substantial healthcare challenge, with little improvement in outcomes since the establishment of the standard of care (SOC) treatment algorithm half a century ago.^[^ ^34^ ^]^ The persistence of recalcitrant biofilm bacteria, manifesting in various forms such as SAC, bacteria within glycocalyx on the implant and necrotic tissue, bacteria within the OLCN of bone, and intracellular bacteria has been identified as the primary cause of clinical failures.^[^ ^1^, ^4^, ^35^ ^]^ Notably, SAC and colonization of OLCN emerge as major contributors to implant failure after revision surgery, as these bacteria can withstand debridement and systemic antibiotic therapy.^[^ ^1^, ^36^ ^]^ This persistence leads to server consequences, including infectious bone destruction, implant failure, and pathological fracture, as evidenced in this study for both the Ti and Ti‐NTs groups without HHC36‐loading (Figure 6D).

Fortunately, the functionalization of titanium implants with antibacterial agents has paved the way for eradicating bacteria through localized release.^[^ ^12^, ^14^, ^18^ ^]^ To address potential cytotoxicity and unnecessary antibacterial therapy, we employed smart stimuli‐responsive strategies, encompassing both exogenous and endogenous stimuli, in the design and preparation of antibacterial‐functionalization of titanium implants.^[^ ^10^, ^15^ ^]^ However, determining the most effective timing of intervention with exogenous stimuli is exceptionally challenging, given that residual bacteria could proliferate at any stage of treatment.^[^ ^19^, ^37^ ^]^

Therefore, infection‐specific endogenous stimuli prove more suitable for achieving bacterial eradication compared to exogenous stimuli. Studies have reported that the acidic byproducts of bacterial infection, such as lactic and acetic acid, can create a local acidic condition (pH values decreasing to 5.0). This phenomenon has been widely employed in developing pH‐responsive antibacterial surfaces for titanium implants.^[^ ^38^ ^]^ In our study, we combined pH‐responsive molecules PMAA, antimicrobial peptides HHC36, and double‐oxidation enhanced TiO_2_ nanotubes to create an endogenous pH‐responsive functionalized surface on the titanium screw (Ti‐NTs‐P‐A). When employed as a device for treating IAO and repairing bone defects, this screw exhibits three key advantages.

The first notable advantage lies in the robustness of the antimicrobial coating on the screw's surface. In clinical applications, such as the insertion of bone screws, considerable torque is required to secure them with the bone. Throughout this process, substantial friction occurs between the bone and the implant surface.^[^ ^39^ ^]^ Many coatings boasting excellent bioactivities, including antibacterial capabilities, often suffer from poor adhesion, leading to noticeable detachment or complete removal and, consequently, a failure to exert effective activity.^[^ ^40^ ^]^ In contrast, the nanotubes fashioned on the screw's surface using the double‐oxidation technology exhibit exceptional mechanical strength.^[^ ^17^, ^41^ ^]^ The results (Figure 2G,H) unequivocally demonstrate their ability to maintain their original controlled release characteristics and sustain over 99% antibacterial activity even after repeated insertion and removals. This underscores the clinical practicality and significance of the Ti‐NTs‐P‐A screw.

The second notable advantage lies in the screw's ability to adapt to various stages post‐implantation. Specifically, it undergoes a burst release within 8 h of implantation, enabling the concentration of AMPs to reach nearly three times the MIC, effectively inhibiting bacteria introduced during the initial implantation.^[^ ^19^, ^42^ ^]^ Subsequently, the release of AMPs transitions into a sustained period, promoting favorable conditions for bone integration.^[^ ^12c^ ^]^ Importantly, in the event of a recurrent bacterial infection, the implant exhibits responsiveness to the decrease in pH, releasing a sufficient amount of AMPs (reaching the MIC within 48 h) (Figure 1I and 2G). This proactive mechanism further contributes to inhibiting bacterial infection.

The third advantage lies in the sustained and controlled release of AMPs facilitated by the gate molecule on this screw, thereby enhancing its antibacterial activity and biocompatibility. In comparison to Ti‐NTs‐A, which lacked gate molecules, Ti‐NTs‐P‐A exhibited a more prolonged release of AMPs. This extended‐release maintains the concentration of AMPs above the MIC for a longer duration, resulting in more effective inhibition of bacterial infection^[^ ^43^ ^]^. This distinction is evident in in vivo antibacterial experiments, where both AMPs‐loaded groups exhibited significant antibacterial activity, Ti‐NTs‐P‐A showed an antibacterial rate of over 98%, surpassing the 81% antibacterial rate of Ti‐NTs‐A (Figure 6C). As it is imperative to eradicate the deep‐seated residual bacteria in the bone tissue to prevent infection recurrence and implant failure, a higher rate of bacterial inhibition is crucial for optimal results.^[^ ^10a,b^ ^]^ This is further emphasized by the results of the BIC, where Ti‐NTs‐P‐A, owing to its superior ability to inhibit bacteria, demonstrates enhanced osteointegration effects compared to Ti‐NTs‐A (Figure 7).

The control of AMPs concentration by Ti‐NTs‐P‐A is superior, preventing excessively high concentrations that could be toxic.^[^ ^12^, ^44^ ^]^ In vitro experiments demonstrated that Ti‐NTs‐A hindered osteogenic differentiation of stem cells due to the burst release of AMPs (Figure 5). Typically, antibacterial materials exhibit poor osteointegration due to the enrichment or burst release of antibacterial agents, which is undesirable but challenging to avoid.^[^ ^45^ ^]^ However, by regulating the release of AMPs through the PMAA gate molecule, Ti‐NTs‐P‐A displays excellent osteointegration effects similar to Ti‐NTs in the absence of infection. Therefore, this strategy offers novel insights for enhancing the biocompatibility of antibacterial materials.

## 4 Conclusion

In conclusion, we have successfully engineered an antibacterial‐functionalized titanium screw (Ti‐NTs‐P‐A) capable of on‐demand release of AMPs from PMAA‐gated TiO_2_ nanotubes for the treatment of IAO. In the physiological environment, PMAA molecules undergo swelling, retaining AMPs within the TiO_2_ nanotubes, thereby enhancing biocompatibility. In acidic infectious environments, PMAA molecules collapse, leading to the abundant release of AMPs peptides from opened nanotubes for effective bacterial eradication.

Critical to this innovation is the demonstrated excellent antibacterial activity and favorable osseointegration of Ti‐NTs‐P‐A screws in infectious conditions, as validated in a rabbit model of implant‐associated *S. aureus* osteomyelitis. Consequently, we propose that pH‐responsive nanotube‐functionalized screws hold great promise for therapeutic applications in osteomyelitis. Furthermore, considering the diversity of primary pathogens in bone infections, further investigations are warranted to assess the therapeutic efficacy of Ti‐NTs‐P‐A screws comprehensively.

## 5 Experimental Section

### Materials

Titanium screw (Ti, 2.7 × 10 mm, WEGO), HHC36 peptides (AMPs, Cys‐Pro‐Ala‐Pro‐Ala‐Pro‐Lys‐Arg‐Trp‐Trp‐Lys‐Trp‐Trp‐Arg‐Arg, 98%, China Peptides Co., Ltd., China), polymethacrylic acid (PMAA, Mw = 9500, Sigma‐Aldrich, Missouri, USA), Phosphate buffered saline (PBS, Gibco, China), Bicinchoninic Acid (BCA) assay kit (Beyotime, China), *S. aureus* (ATCC29213), LIVE/DEAD backlight bacterial viability kit (Solarbio, China), Cell Counting Kit‐8 (CCK‐8) (Dojindo, Japan).

### Screw Surface Modification

pH‐responsive nanotubes on the screw were prepared as previously reported.^[^ ^12^, ^17^ ^]^ Briefly, Ti screws were treated with 3 vol% HF solution for 1 min and cleaned with deionized water. Ti screws (anode) and platinum foil (cathode) were incubated with electrolyte solution (0.5 wt% NH_4_F and 1 m (NH_4_)_2_SO_4_) at 20 V for 30 min (named Ti‐NTs‐ctrl). Then an additional anodization (20 V, 5 min) was performed in 1m (NH_4_)_2_SO_4_ electrolyte. After the double anodization process, the obtained screws were washed with deionized water and denoted Ti‐NTs. Then, Ti‐NTs screws were treated with oxygen plasma for 5 min and Tris‐buffer (pH 8.5) containing 3,4‐dihydroxyphenethylamine hydrochloride (dopamine, 1 mg mL^−1^) for 24h sequentially, then denoted Ti‐NTs‐D. For grafting PMAA molecules onto the nanotubes, the Ti‐NTs‐D screws were immersed in a reaction solution containing 1‐ethyl‐3‐(3‐dimethylaminopropyl)‐carbodiimide hydrochloride (EDC, 50 mm), N‐hydroxysuccinimide (NHS, 20 mm) and PMAA (5 mm) for 24 h. After deionized water cleaning, the obtained screws were denoted by Ti‐NTs‐P.

### Screw Surface Characterization

Microscopic images of the prepared screws were obtained by scanning electron microscope (SEM, Quanta 200, FEI). XPS (Kratos Axis Ultra, UK) was performed to confirm the chemical composition of the screws following adonization, dopamine binding, and PMAA binding. To measure the adhesion strength between the synthesized titanium dioxide nanotubes and Ti substrate, scratch tests of Ti‐NTs and Ti‐NTs‐ctrl (without additional oxide layer) were performed by using an auto scratch tester (STEP 700 Anton Paar, Austria). The critical load (L_c_), the smallest load at which a recognized coating layer failed, was determined from the load versus acoustic signal.

### HHC36 Peptides Loading and Release of the Functionalized Screws

For HHC36 loading, a 1 mmol L^−1^ HHC36 solution was prepared in ethanol, and the pH was adjusted to 5.0 using HCl to collapse the PMAA molecules. Then, 25 µL HHC36 solution was added to the screws (Ti‐NTs and Ti‐NTs‐P) in a 24‐well plate, and the screws were dried under a vacuum desiccator. This loading process was repeated for a total of 9 times. For in vitro and in vivo antibacterial ability testing, the obtained samples were sterilized by steam under high pressure and were denoted as Ti‐NTs‐A and Ti‐NTs‐P‐A, respectively. To determine the HHC36 loading content, the Ti‐NTs‐A and Ti‐NTs‐P‐A screws were washed with 1 mL PBS solution 3 times, and the HHC36 loading content of each screw was quantified with a BCA kit as follows:

(1) $$\begin{array}{ccc} \mathrm{Loading}\;\mathrm{content} & & =\mathrm{total}\;\mathrm{added}\;\mathrm{content}\;-\;\mathrm{remaining}\;\mathrm{content}\;\\ & & \;\left(\mathrm{in}\;\mathrm{PBS}\;\mathrm{solution}\right) \end{array}$$

For the HHC36 release behavior test, Ti‐NTs‐A or Ti‐NTs‐P‐A screws were placed in 24‐well plates with 500 µL of PBS (pH 5.0 or 7.4) in each well. The plates were then shaken at 150 rpm, 37 °C. At predetermined time points, the incubation solution was collected, and the concentration of HHC36 peptide released from different screws was determined by BCA assay. During the release process, the pH of the solution was adjusted from 7.4 to 5.0 at 96 h for 48 h to simulate an infectious environment.

### In Vitro and Ex Vivo Bactericidal Assay of Functionalized Screws

For analysis of repeated bactericidal activity, screws with or without HHC36‐loading were placed into a 24‐well plate, and 500 µL *S. aureus* (ATCC29213) bacterial suspension (1 × 10^6^ CFU mL^−1^) was added to fully cover the screws. After shaking at 150 rpm, 37 °C for 1 h, the bacterial suspension was taken for CFU counts. Then, the screws were air‐dried, and the above procedures were repeated 3 more times.

As mentioned above, the incubation solutions from Ti‐NTs, Ti‐NTs‐A, and Ti‐NTs‐P‐A screws were obtained after 12 h incubation. Then, 500 µL bacteria suspension (*S. aureus*, 2 × 10^6^ CFU mL^−1^) in Luria‐Bertani (LB) Medium was mixed with 500 µL incubation solution from each Ti‐NTs, Ti‐NTs‐A or Ti‐NTs‐P‐A screw, and shaken at 150 rpm, 37 °C for 2 h. Serial dilutions of each co‐incubation were plated to an LB agar plate incubated at 37 °C overnight for CFU counts. In addition, bacterial viability on the screws’ surfaces was assessed by co‐culturing the screws and bacteria (250 µL, 1 × 10^6^ CFU mL^−1^) for 4h, followed by visualization using Live/Dead backlight bacterial viability kits and confocal laser scanning microscopy (CLSM, Andor Dragonfly 200, UK).

To test the stability of the prepared screws, abrasion tests were conducted according to the clinical implantation process of orthopedic screws. Briefly, a cylindrical defect (2.5 mm in diameter × 8 mm in depth) was made on rabbit tibial plateau using a low‐speed spherical grinding drill under continuous saline irrigation, and the screws were screwed into the defects. Then, the screws were extracted and washed gently with PBS for SEM analysis. HHC36 release profiles and antibacterial abilities of the unscrewed screws were also investigated using the same protocols as mentioned above.

To observe the morphology of *S. aureus* on different screws, the Ti, Ti‐NTs, Ti‐NTs‐A, or Ti‐NTs‐P‐A screw was incubated with *S. aureus* (1 × 10^6^ CFU mL^−1^ in LB medium, 500 µL) at 150 rpm, 37 °C for 4 h. Then, the screws were collected and fixed in glutaraldehyde (2.5%, w/v) for 24 h. After gradient dehydration, the specimens were dried in a critical point dryer, coated with gold‐palladium, and then observed using a Hitachi Su‐8010 SEM (Hitachi, Japan).

### Transcriptome Sequencing and Data Analysis

500 µL bacteria suspension (*S. aureus*, 1 × 10^7^ CFU mL^−1^), diluted in LB medium, was incubated with or without a Ti‐NTs‐P‐A screw at 150 rpm, 37 °C for 3 h. Then the cells were collected by centrifugation at 10 000 rpm for 10 min, and the total RNA of *S. aureus* was extracted using TRIzol reagent (Guangzhou IGE Bio, China) according to the protocols provided by the manufacturer. RNA purification, sequencing, and data analysis were performed by Guangzhou IGE Biotechnology (China). Three independent assays were performed for each group. The thresholds for significant differential expression were |log_2_(fold change)| ≥ 1 and false discovery rate (FDR) < 0.05.

### Cell Culture

Multipotent mouse embryonic fibroblasts (MEFs), obtained from the Molecular Oncology Laboratory, University of Chicago Medical Center, USA., were applied for the cell viability and osteogenic differentiation assays as previously reported.^[^ ^26^, ^46^ ^]^ Cell culture medium was obtained by supplementing Dulbecco's Modified Eagle Medium (DMEM) with 10% fetal bovine serum and 1% penicillin‒streptomycin. For the osteoblastic differentiation assay, an osteoblastic induction medium (OBM), containing 50 nm ascorbic acid (Sigma, USA), 10 mm β‐sodium glycerophosphate (Solarbio, China), and 0.1 µm dexamethasone (Sigma, USA), was prepared from the cell culture medium.

The Ti, Ti‐NTs, Ti‐NTs‐A, or Ti‐NTs‐P‐A screw was placed in 1 mL cell culture medium or OBM, and incubated at 150 rpm, 37 °C for 12 h. Then, the cell culture medium and OBM were collected and sterilized with 0.22 µm syringe filters (Millex) for cytocompatibility and osteoblastic differentiation assay, respectively.

### Cellular Compatibility Assay

MEFs were seeded into 48‐well plates (1 × 10^4^ cells per well) and cultured at 37 °C under 5% carbon dioxide (CO_2_) for 24 h. Then, the cell culture medium was replaced with the incubation medium. In the control group, the MEFs were always cultured with a cell culture medium. The medium in both the experimental group and control group was replaced every 2 days. At predetermined time points, cell viability was investigated by Cell Counting Kit‐8 (CCK‐8) (Dojindo, Japan) according to the manufacturer's instructions.

### In Vitro Osteoblastic Differentiation Assay

MEFs (1 × 10^4^ cells per well) were seeded into 24‐well plated and cultured overnight. Then the culture medium was changed to an incubation medium (obtained by immersing screws with OBM) in the Ti, Ti‐NTs, Ti‐NTs‐A, and Ti‐NTs‐P‐A groups for osteoblastic differentiation assay. In the control and OBM groups, cells were cultured with cell culture medium and OBM, respectively. An Alkaline phosphatase (ALP) assay kit (Beyotime, China) was utilized to determine the ALP activity in the cell supernatant as instructed by the manufacturer. For western blot analysis, the protein was extracted from the cells at the designated time points and separated via SDS‐PAGE. Then, the gels were transferred to polyvinylidene difluoride (PVDF) membranes (0.22 µm, Millipore, USA). The target proteins, including RUNX2 (Abmart, dilution 1:1000), OSTERIX (Abmart, dilution 1:1000), COL1 (Abmart, dilution 1:1000), OCN (Abmart, dilution 1:1000), and GAPDH (Servicebio, dilution 1:3000) were detected using specific antibodies. Additionally, the MEFs treated as described above were fixed with 4% polyformaldehyde for 15 min and permeabilized with 0.1% Triton‐X for 15 min. After incubated with primary antibody RUNX2 (Abmart, dilution 1:500) or COL1 (Abmart, dilution 1:500) at 4 °C overnight, the MEFs were exposed to secondary antibody (Servicebio, dilution 1:500) for one hour at room temperature. Nuclei were visualized by 4,6 diamidino‐2‐phenylindole (DAPI) staining for 5 min, followed by three washes with PBS. The stained cells were then observed and photographed via a fluorescence microscope (Olympus BX53F, Japan).

### In Vivo Osseointegration of Functionalized Screws in Non‐Infected Model

All animal procedures were approved by the institutional ethics committee (IEC) of Chongqing Medical University (No: IACUC‐CQMU‐2023‐0277). 12 to 14‐week‐old male New Zealand white rabbits (2–2.5 kg body weight) were used for the experiment. Food and drink were withheld for 6 h before anesthesia for all experimental rabbits. After anesthesia via intravenous injection of pentobarbital (25mg kg^−1^), the skin on the knee was prepared. The rabbits were randomized into four groups, where Ti, Ti‐N, Ti‐N‐ A, and Ti‐N‐P‐A screws were implanted into the left tibia of rabbits under sterile conditions.

After 8 weeks, the rabbits were sacrificed, and tibia samples were collected and fixed in 4% paraformaldehyde for further analysis. A micro‐CT system (µCT100, Scanco Medical, Switzerland) was used to scan the samples, and quantitative analysis (bone tissue volume/total tissue volume, BV/TV) and 3D reconstruction of the new bone surrounding the screws were carried out. For non‐decalcified sections, the tibia implanted with the screw was embedded in methyl methacrylate after gradient alcohols and xylene treatment. Then, hard tissue sections with a thickness of ≈20 µm were prepared by cutting and grinding and stained with methylene blue‐basic magenta. Image J software was used to quantitively analyze the bone‐implant contact (BIC) ratio.

To test the in vivo biocompatibility of the Ti‐NTs‐P‐A screw, the major organs (heart, liver, spleen, lung, and kidney) of the experimental rabbits were collected for pathological analysis using H&E staining. Moreover, blood samples were collected and biochemistry analysis was conducted using a biochemical automated analyzer (chemray 800, China).

In vivo antibacterial ability and osseointegration of functionalized screws in implant‐associated S. aureus osteomyelitis model: The experimental rabbits were prepared and anesthetized before surgery as mentioned above. The bacterial solution (*S. aureus* in the logarithmic phase of growth, 3 × 10^10^ CFU mL^−1^) was used to contaminate sterile titanium screws overnight. Subsequently, these bacteria‐loaded screws were air‐dried for 30 min in a sterile laminar flow hood, and the bacterial load of each screw was determined almost 5 × 10^7^ via CFU assay as described in the previous publications.^[^ ^5c^ ^]^ Then, these screws were implanted into the left tibiae of rabbits. The surgical sites were then layer‐sutured and remained untreated for 1 week to establish the IASO model.

After the 7 days of the infection phase, the rabbits were randomized into four groups (*n* = 8 per group), and 1‐stage revision surgery was performed. Concisely, the implanted screw was removed and followed by wound debridement and saline irrigation. Then, different types of screws were implanted as a replacement for an additional 4 weeks of follow‐up. After 4 weeks, the rabbits were euthanized and the tibiae in each group were harvested for CFU assay (*n* = 5). The obtained screws were placed in a Falcon tube with PBS solution and sonicated for 15 min, and vortexed for 2 min. The serial dilutions of each specimen were plated to LB agar plates and incubated at 37 °C overnight for CFU counts. Three specimens in each group were fixed in 4% paraformaldehyde for 48 h and subjected to micro‐CT and 3D reconstruction analysis. The BV/TV of the new bone surrounding the screws was quantified. In addition, 20‐µm hard tissue sections were stained with methylene blue‐basic magenta for BIC assays. Other tibial specimens were decalcified, dehydrated, embedded in paraffin, and made into 5 µm sections for H&E and Gram staining.

### Statistical Analysis

All data were statistically analyzed using GraphPad Prism 8.0 Software. Values are presented as the means ± standard deviations (SDs). An independent‐sample *t*‐test and one‐way ANOVA were used for intergroup comparisons. Statistical significance: ^*^ *p* < 0.05, ^**^ *p* < 0.01, ^***^ *p* < 0.001, and ^#^ *p* < 0.05, ^##^ *p* < 0.01, ^###^ *p* < 0.001.

### Ethics Approval and Consent to Participate

Research involving animals was approved by the institutional ethics committee (IEC) of Chongqing Medical University (approval number: IACUC‐CQMU‐2023‐0277). All animal housing and experiments were conducted in strict accordance with the institutional guidelines for the care and use of laboratory animals.

## Conflict of Interest

The authors declare no conflict of interest.

## Supporting information



Supporting Information

## Data Availability

The data that support the findings of this study are available from the corresponding author upon reasonable request.
