# Supplementary material for: Efficacy of pH‐Responsive Surface Functionalized Titanium Screws in Treating Implant‐associated S. aureus Osteomyelitis with Biofilms Formation
Source: Adv Healthc Mater. 2024 Nov 27;14(3):2403261. doi: 10.1002/adhm.202403261 (PMC11773098; doi:10.1002/adhm.202403261)
Supplement: Supplementary file 1 — Supporting Information [file ADHM-14-0-s001.docx]

**Efficacy of pH-responsive Surface Functionalized Titanium Screws in Treating Implant-associated *S. aureus* Osteomyelitis with Biofilms Formation**

*Hang Zhou, Youliang Ren, Kaixiong Zou, Ying Jin, Hang Liu, Haitao Jiang, Lei Shi, Xiaomin Sheng, Jason Weeks, Hannah Wang, Thomas Xue, Edward M. Schwarz, Chao Xie, Zhongliang Deng^*^, Lin Wang^*^, Lei Chu^*^*

**Supporting Information**


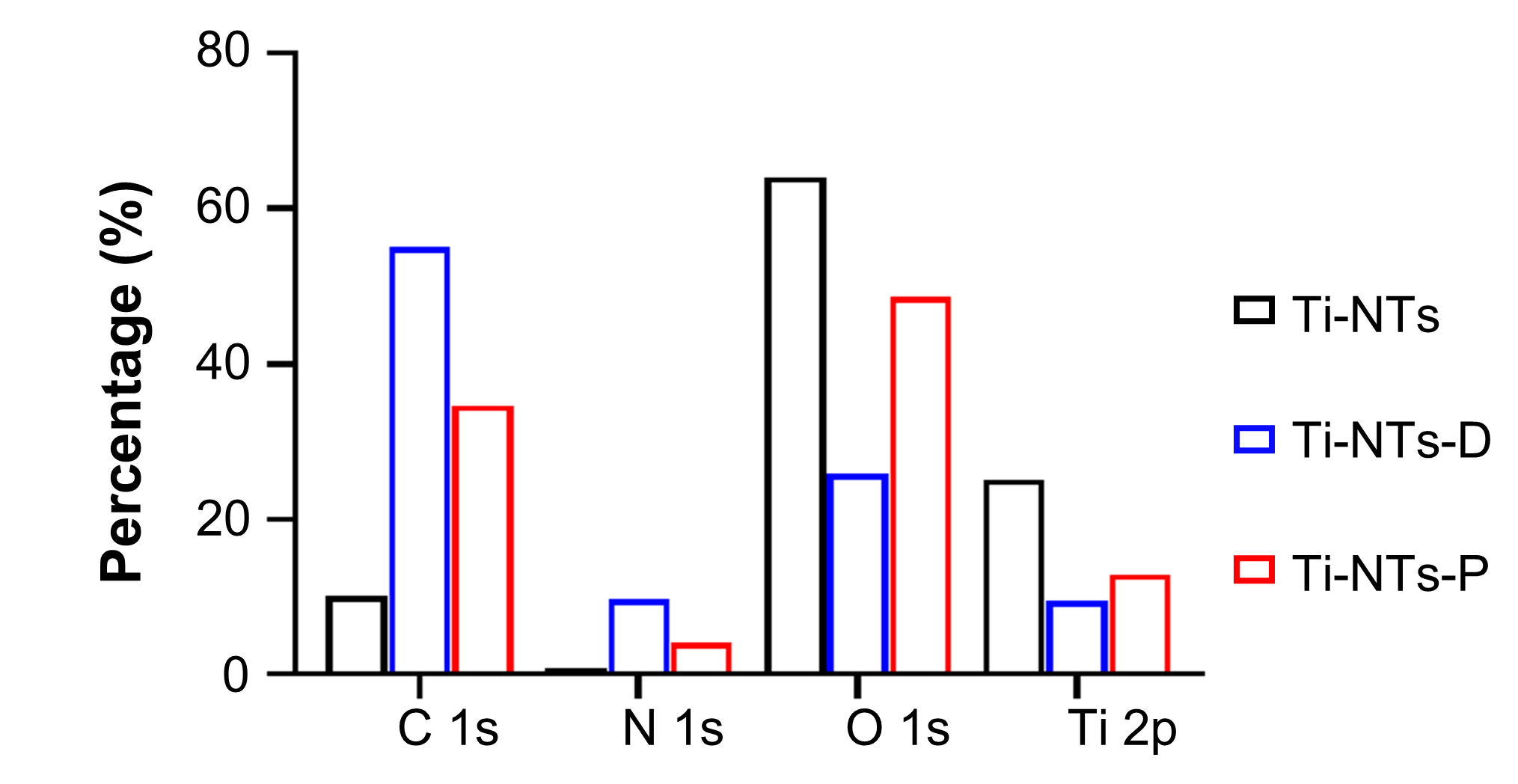


**Figure S1.** The elemental compositions of the indicated screws determined by XPS.


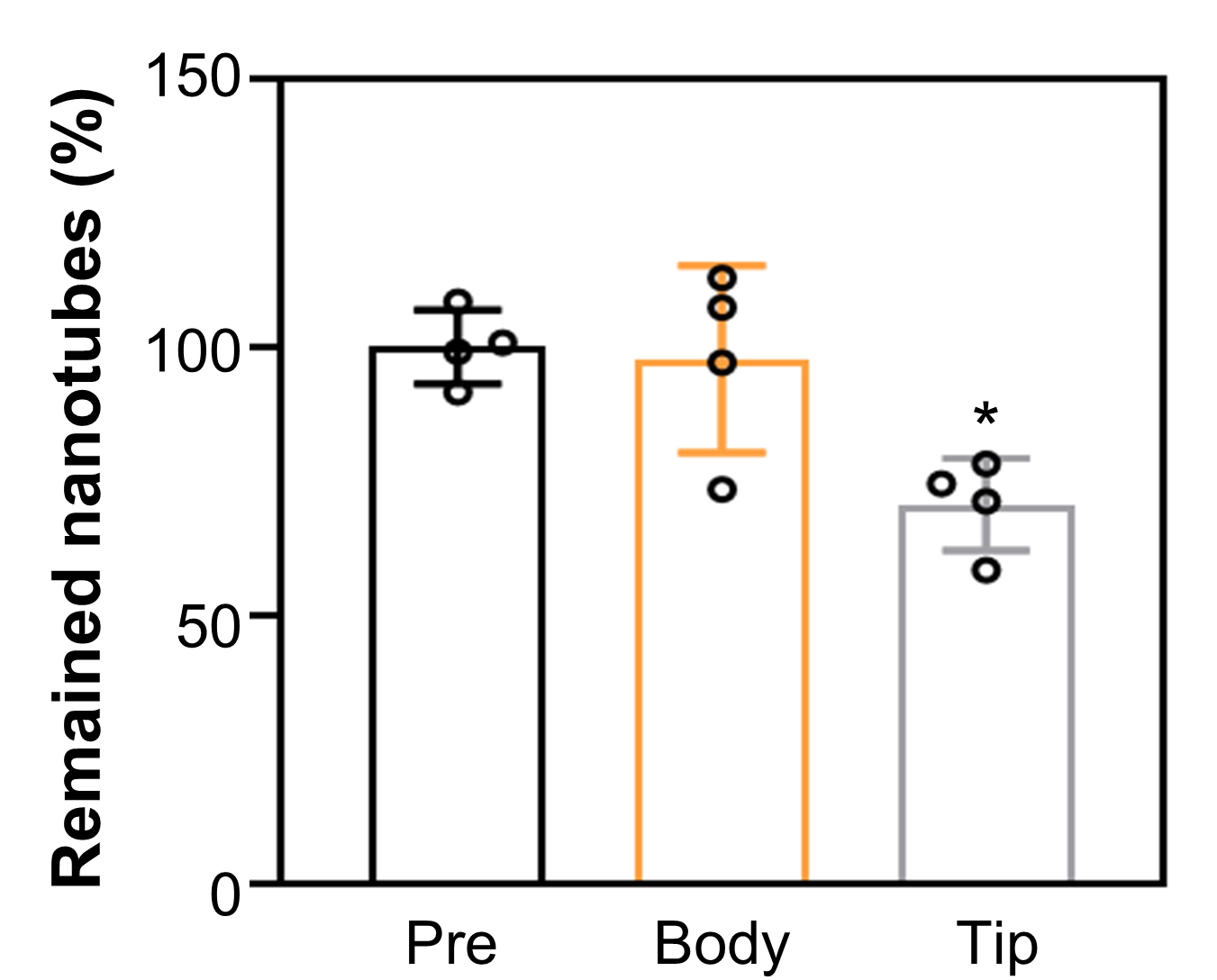


**Figure S2.** The quantitative analysis of the remaining nanotubes on the functional screws after implantation (n = 4). The data is shown as the means ± SDs, * *p* < 0.05, in comparison with the pre group. Pre, pre-implantation. Body, screw body. Tip, screw tip.


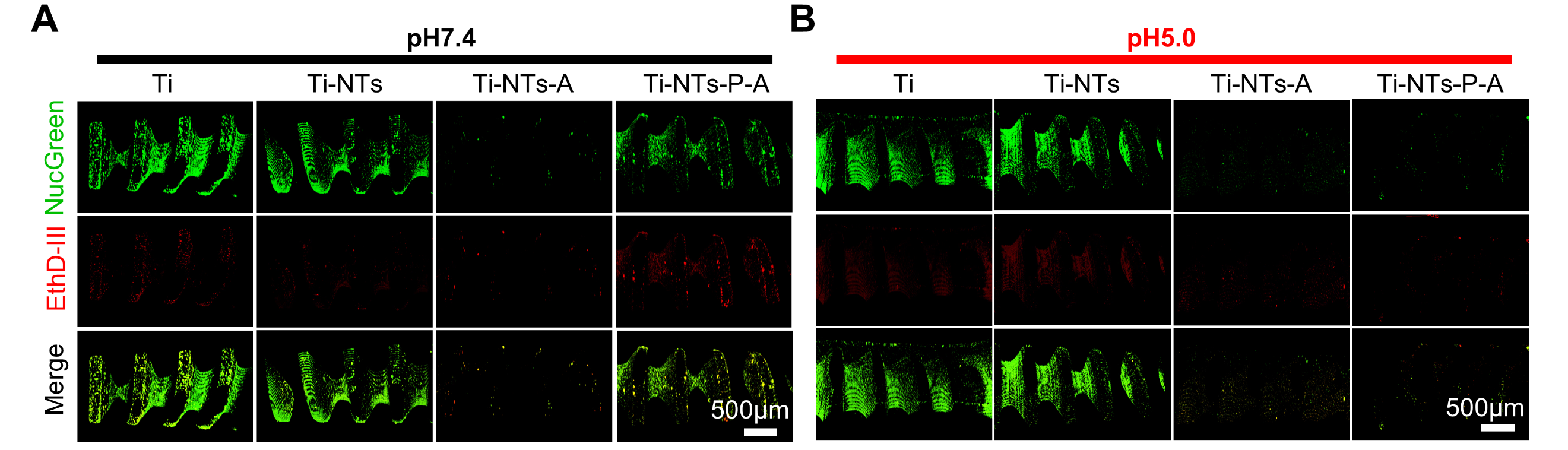


**Figure S3.**3D reconstructed fluorescence images of live/dead stained *S. aureus* after incubation with the indicated unscrewed screws for 4 h (Green, live and dead bacteria; Red, dead bacteria).


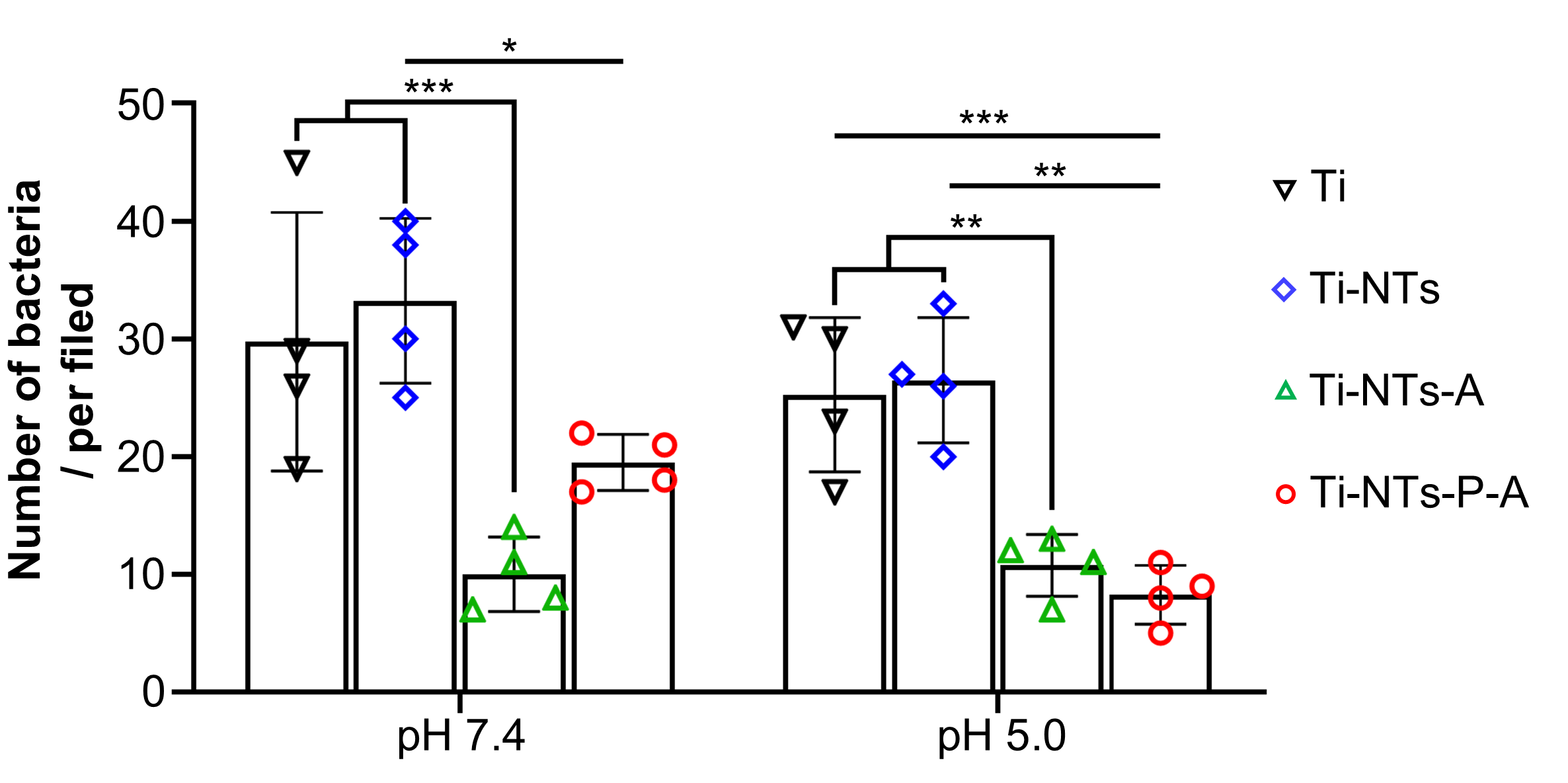


**Figure S4**. The quantitative analysis of the adherent bacteria in random field (n = 4). The data is shown as the means ± SDs, * *p* < 0.05, ** *p* < 0.01, *** *p* < 0.001.


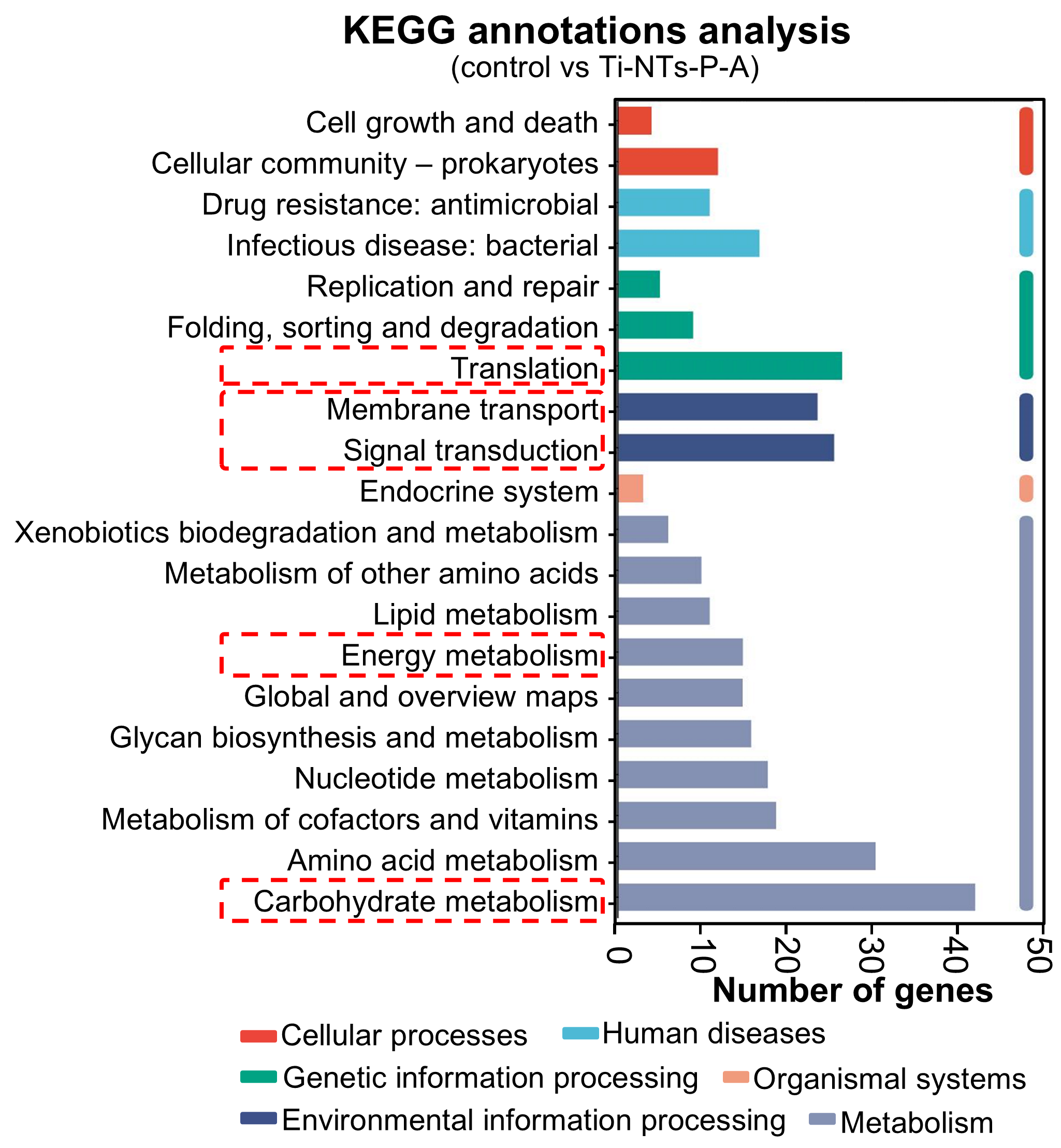


**Figure S5.** KEGG annotations analysis of the significantly differentially expressed genes between the control and the Ti-NTs-P-A groups.


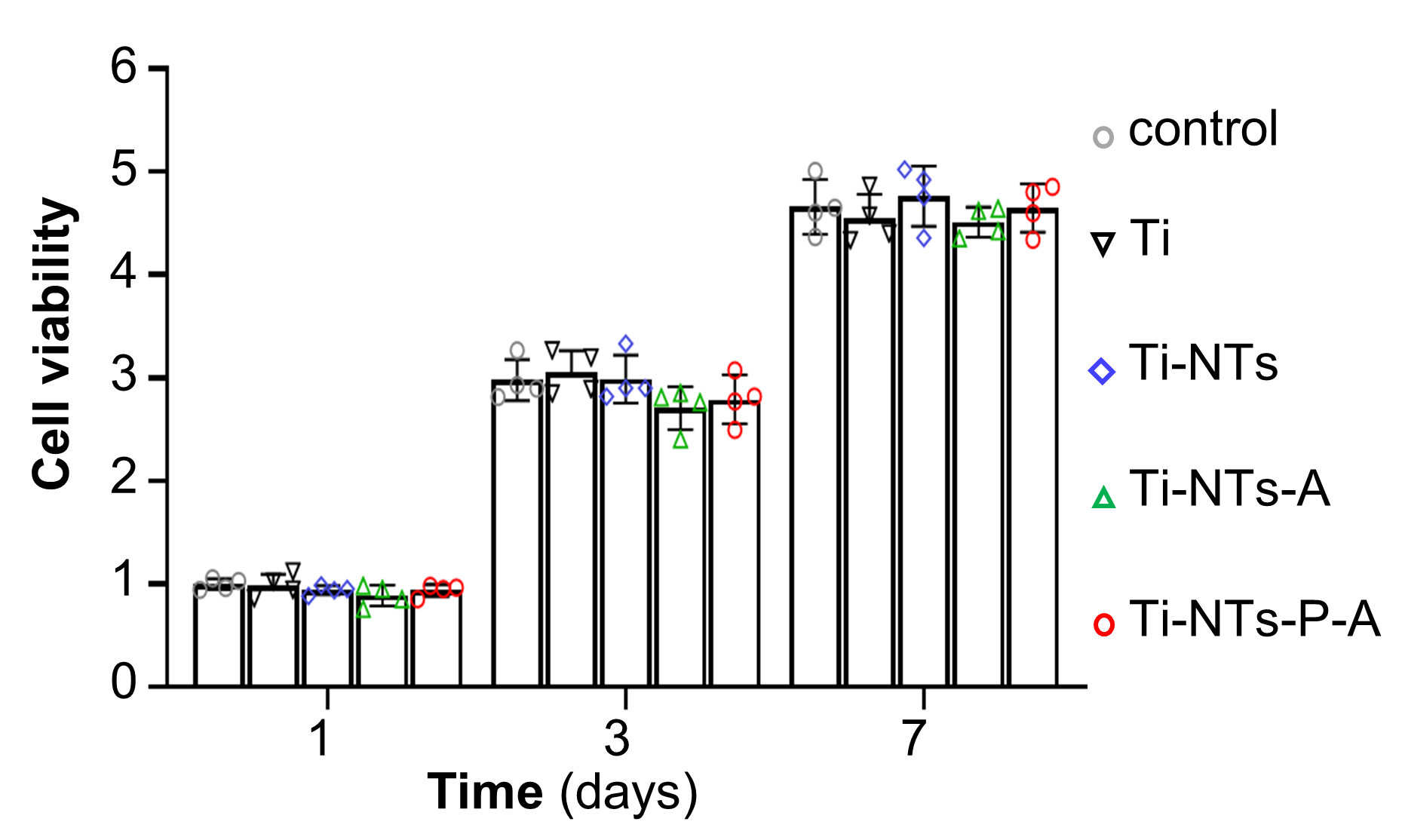


**Figure S6**. CCK-8 results were obtained after culturing MEFs in incubation solution from different screws for 7 days (n = 4). Data are shown as the means ± SDs.


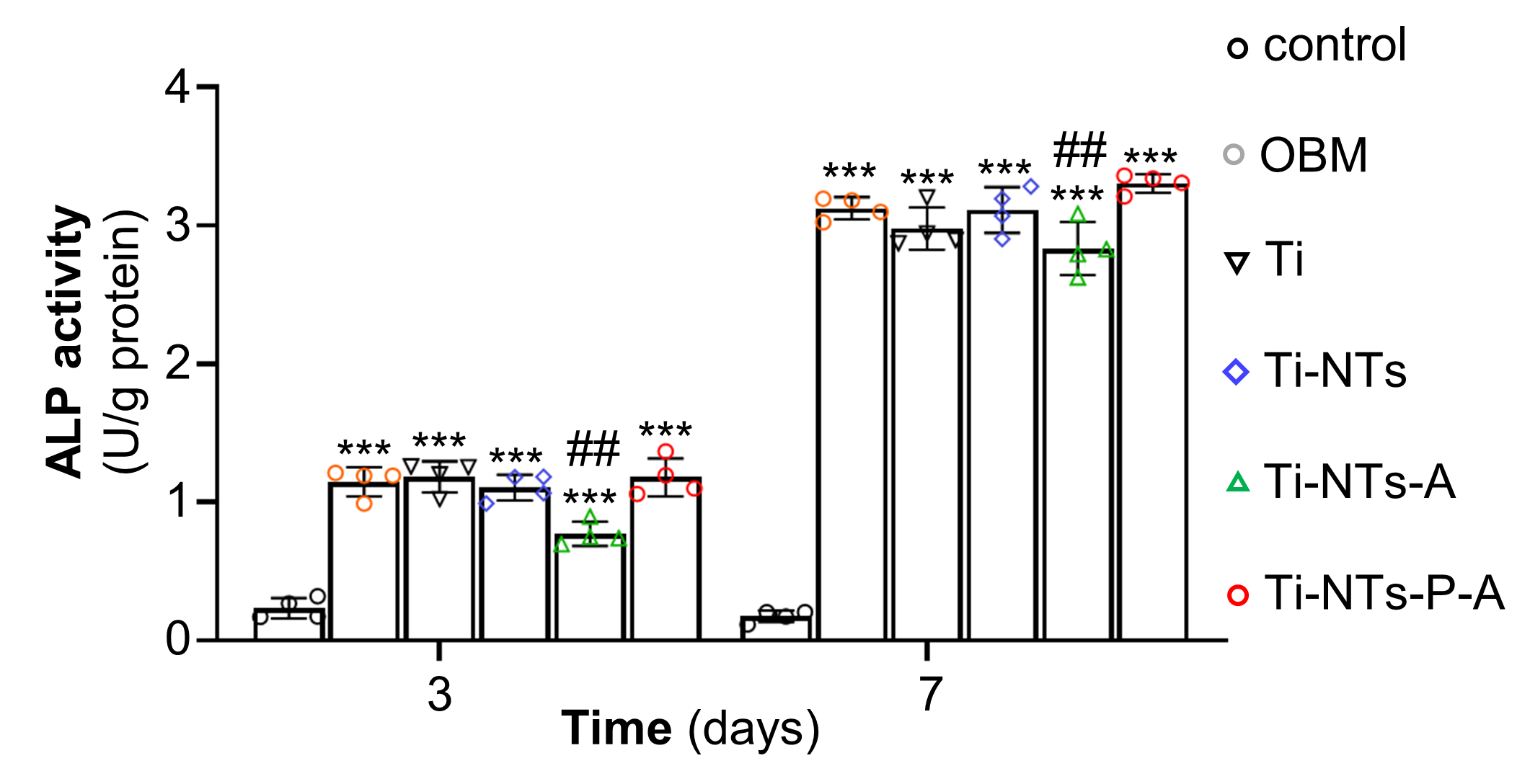


**Figure S7.** The ALP activities of the MEFs treated with different incubation solution (n = 4). Data are shown as the means ± SDs, *** *p* < 0.001, in comparison with the control group, ^###^ *p* < 0.001 in comparison with the OBM group.


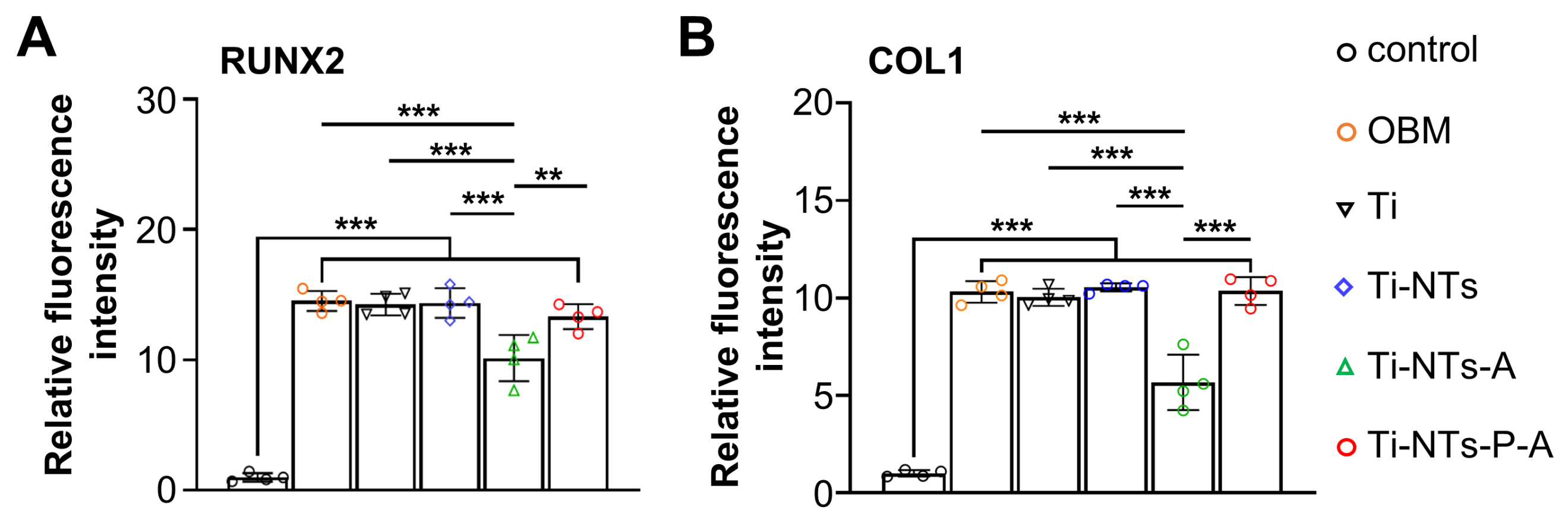


**Figure S8.** The relative fluorescence intensity quantification of RUNX2 and COL1 (n = 4). The data are expressed as the mean ± SD. ***p* < 0.01 and ****p* < 0.001


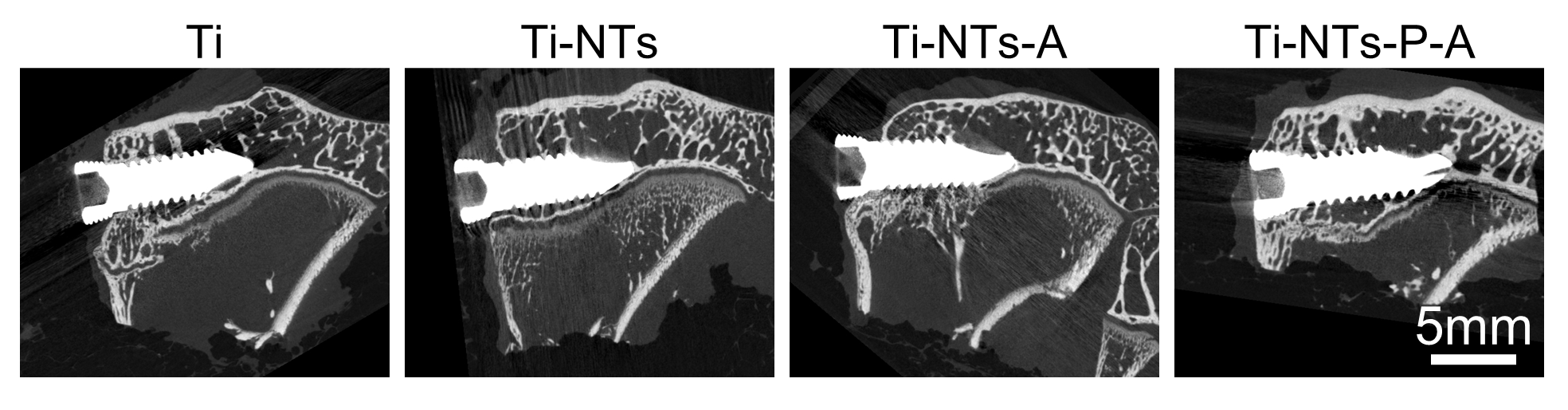


**Figure S9**. The reconstructed micro-CT images of the rabbit tibia 56 days post-implantation.


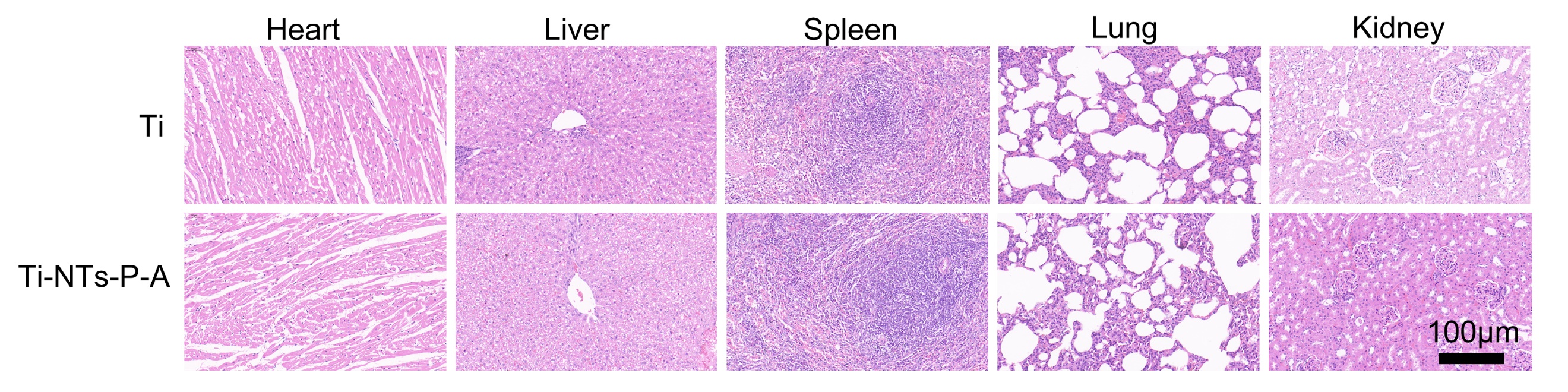


**Figure S10.** H&E staining of heart, liver, spleen, lung and kidney after 56 days of implantation. No pathological changes were observed in all groups.


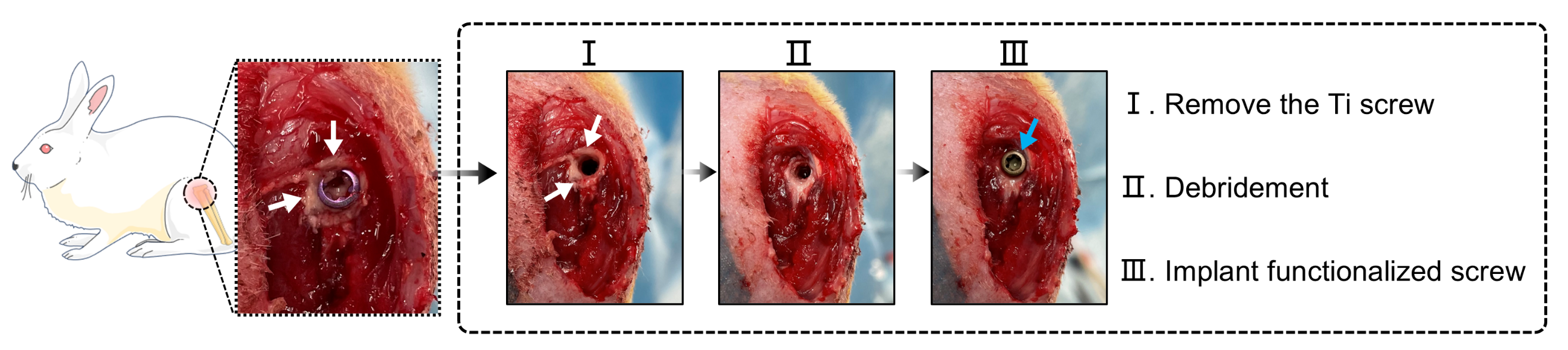


**Figure S11**. The process of the rabbit tibia one-stage revision model. After a 7-days infection phase, the Ti screw was unscrewed. The indicated functionalized screws (blue arrow) were implanted as a replacement after the debridement of the infected tissue (white arrow).


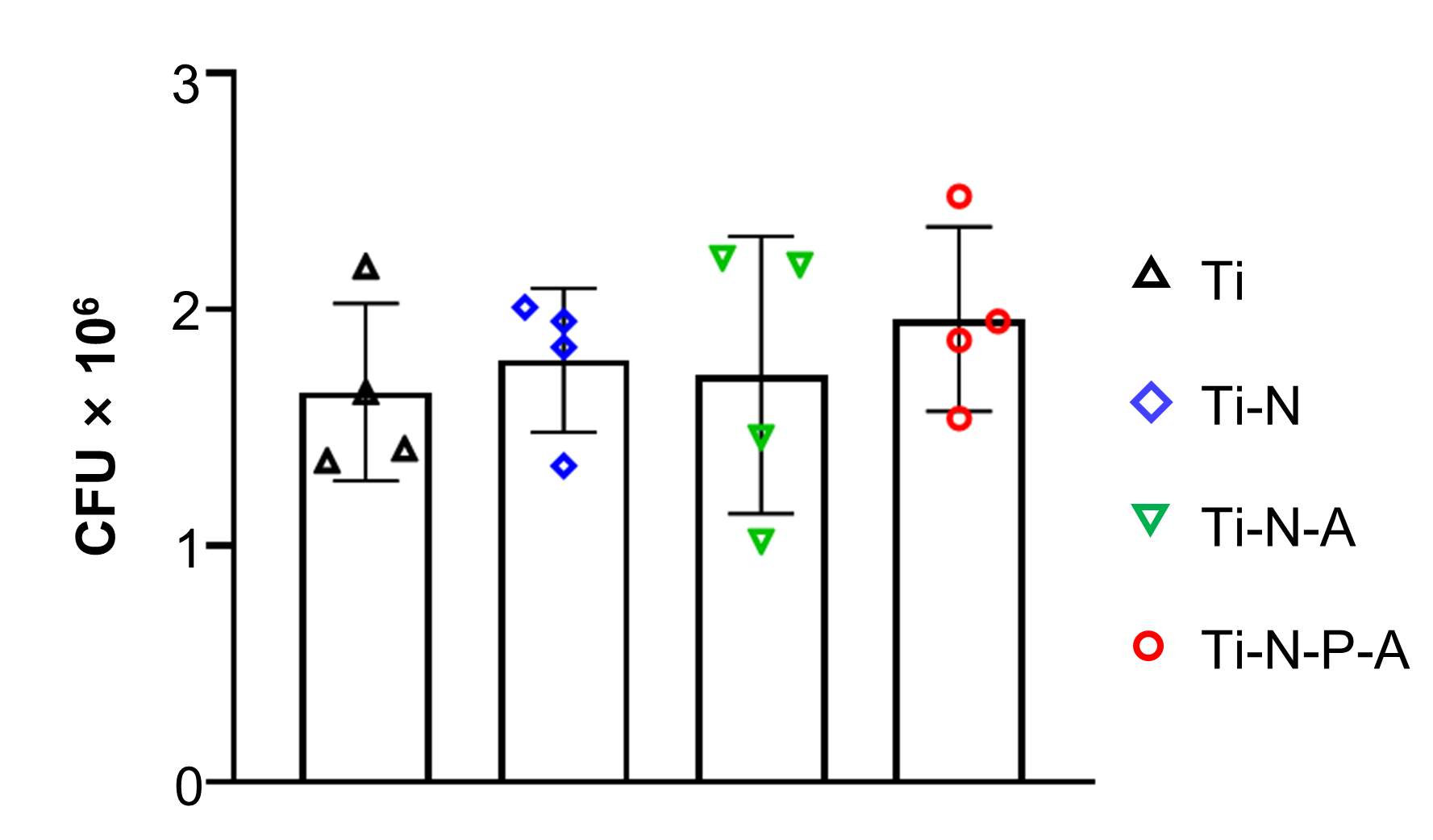


**Figure S12**. The CFU assay of unscrewed screws in different groups after 7 days of infection stage (n = 4). Data are shown as the means ± SDs.


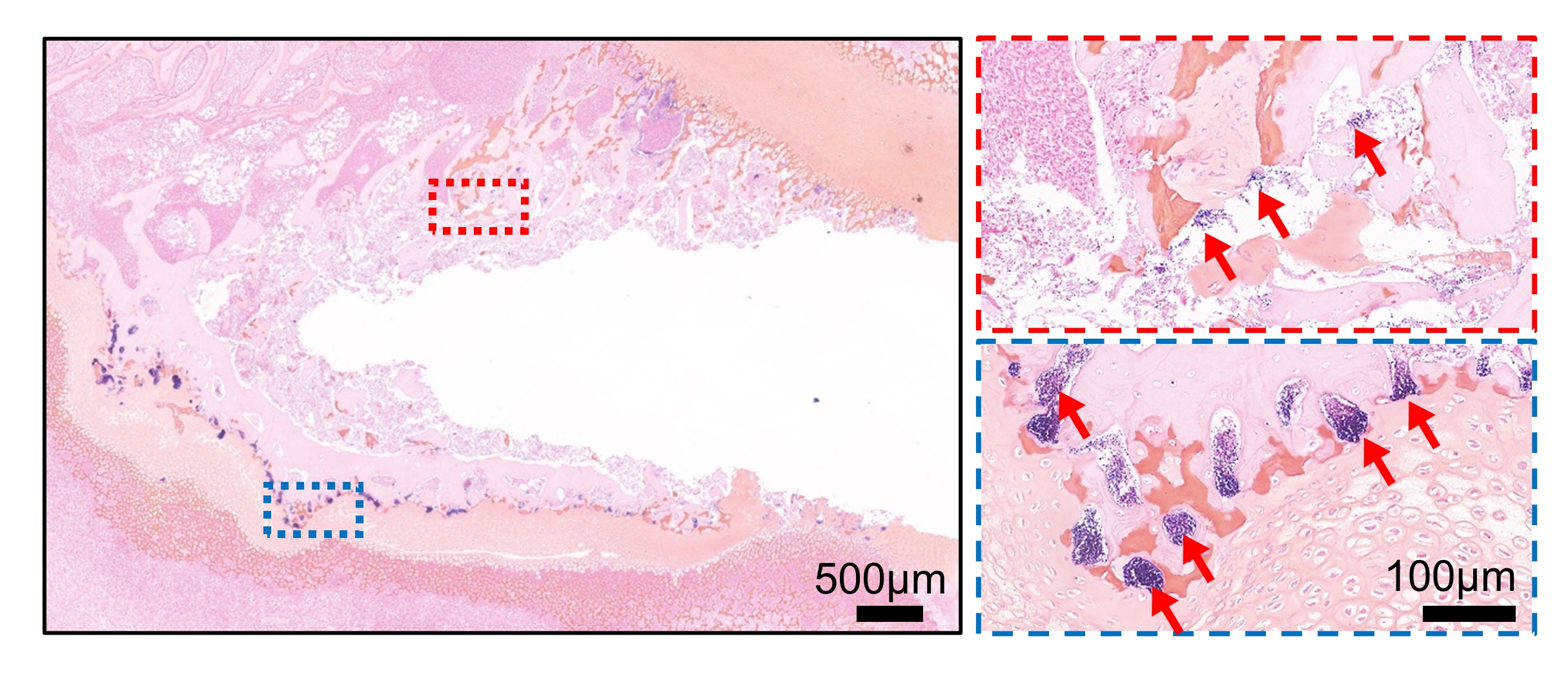


**Figure S13**. Representative images of Gram-positive bacteria within rabbit tibia after 7 days of infection. Red arrows indicated Gram-positive bacteria.

|  | **ALT**  (U/L) | **AST**  (U/L) | **BUN**  (mmol/L) | **CREA**  (μmol/L) |
| --- | --- | --- | --- | --- |
| **Reference rages** | 10-100 | 10-100 | 3-11 | 20-150 |
| **Ti** | 38.3 ± 11.3 | 39.1 ± 9.3 | 6.0 ± 0.2 | 66.7 ± 17.6 |
| **Ti-NTs-P-A** | 34.3 ± 8.2 | 36.3 ± 7.7 | 6.2 ± 1.3 | 62.7 ± 20.2 |

**Table S1.** The serological analysis of the Ti and Ti-NTs-P-A groups after 56 days of screws implantation (n = 3). All results were among the normal reference values.
